# *In vitro* safety and functional characterization of the novel *Bacillus coagulans* strain CGI314

**DOI:** 10.3389/fmicb.2023.1302480

**Published:** 2024-01-10

**Authors:** Shahneela Mazhar, Annie Simon, Ekaterina Khokhlova, Joan Colom, Natasha Leeuwendaal, John Deaton, Kieran Rea

**Affiliations:** ^1^ADM Cork H&W Limited, Bio-Innovation Unit, University College Cork, Cork, Ireland; ^2^ADM Deerland Probiotics and Enzymes, Kennesaw, GA, United States

**Keywords:** *Bacillus coagulans* CGI314, probiotic properties, functional foods, antioxidant, intestinal-epithelial barrier integrity

## Abstract

**Introduction:**

*Bacillus coagulans* species have garnered much interest in health-related functional food research owing to their desirable probiotic properties, including pathogen exclusion, antioxidant, antimicrobial, immunomodulatory and food fermentation capabilities coupled with their tolerance of extreme environments (pH, temperature, gastric and bile acid resistance) and stability due to their endosporulation ability.

**Methods:**

In this study, the novel strain *Bacillus coagulans* CGI314 was assessed for safety, and functional probiotic attributes including resistance to heat, gastric acid and bile salts, the ability to adhere to intestinal cells, aggregation properties, the ability to suppress the growth of human pathogens, enzymatic profile, antioxidant capacity using biochemical and cell-based methods, cholesterol assimilation, anti-inflammatory activity, and attenuation of hydrogen peroxide (H_2_O_2_)-induced disruption of the intestinal-epithelial barrier.

**Results:**

*B. coagulans* CGI314 spores display resistance to high temperatures (40°C, 70°C, and 90°C), and gastric and bile acids [pH 3.0 and bile salt (0.3%)], demonstrating its ability to survive and remain viable under gastrointestinal conditions. Spores and the vegetative form of this strain were able to adhere to a mucous-producing intestinal cell line, demonstrated moderate auto-aggregation properties, and could co-aggregate with potentially pathogenic bacteria. Vegetative cells attenuated LPS-induced pro-inflammatory cytokine gene expression in HT-29 intestinal cell lines and demonstrated broad antagonistic activity toward numerous urinary tract, intestinal, oral, and skin pathogens. Metabolomic profiling demonstrated its ability to synthesize several amino acids, vitamins and short-chain fatty acids from the breakdown of complex molecules or by *de novo* synthesis. Additionally, *B. coagulans* CGI314’s strong antioxidant capacity was demonstrated using enzyme-based methods and was further supported by its cytoprotective and antioxidant effects in HepG2 and HT-29 cell lines. Furthermore*, B. coagulans* CGI314 significantly increased the expression of tight junction proteins and partially ameliorated the detrimental effects of H_2_O_2_ induced intestinal-epithelial barrier integrity.

**Discussion:**

Taken together these beneficial functional properties provide strong evidence for *B. coagulans* CGI314 as a promising potential probiotic candidate in food products.

## 1 Introduction

Novel thermostable and acid-tolerant probiotic spores are an emerging concept in developing functional foods, specifically in the manufacture of processed foods offering superior health benefits over native foods (Guimarães et al., 2020; Poshadri et al., 2022). *Bacillus* strains are widespread in nature and are found in air, soil, fermented foods, and the human gut (Casula and Cutting, 2002; Cutting, 2011; Elshaghabee et al., 2017). Beneficial qualities of *Bacillus* probiotics include their resistance to high temperatures, UV radiation, desiccation, and tolerance to low pH and high concentrations of bile salts due to their spore-forming abilities. It has been demonstrated that spores of *Bacillus* probiotics germinate, grow, and re-sporulate in the gastrointestinal tract (Casula and Cutting, 2002; Colom et al., 2021). The current commercial *Bacillus* probiotic strains include *Bacillus cereus, Bacillus clausii*, *Bacillus coagulans*, *Bacillus indicus*, *Bacillus licheniformis*, *Bacillus polyfermenticus*, *Bacillus pumilus*, and *Bacillus subtilis* (Lee et al., 2019; Saggese et al., 2021). Amongst the *Bacillus* species, *Bacillus coagulans* are characterized as atypical members, as they differ in several physiological, and biochemical characteristics, including the ability to produce high amounts of lactic acid from the fermentation of glucose, sucrose, maltose, and mannitol; thus were formerly classified as *Lactobacillus sporogenes* (De Clerck et al., 2004; Konuray and Erginkaya, 2018; Cao et al., 2020).

*Bacillus coagulans* is a gram-positive, facultative anaerobic, nonpathogenic, endospore-forming, lactic acid-producing bacteria and has been granted Generally Recognized as Safe (GRAS) status by the US Food and Drug Administration (FDA) (Cao et al., 2020). Several studies have demonstrated the beneficial effects of this probiotic species when administered as a feed additive or dietary supplement to livestock, aquaculture and human health (Hung et al., 2012; Gu et al., 2015; Izadi et al., 2020; Lu et al., 2023). In preclinical studies, *B. coagulans* has demonstrated improved body weight, enhanced average daily gain, improved feed conversion ratio, antioxidant capacity, immunity function and gut health in broiler chickens (Hung et al., 2012; Zhang et al., 2021; Cao et al., 2022). In several human studies different strains of this species displayed beneficial effects for managing symptoms of gastrointestinal distress in adults and the pediatric population (Sudha et al., 2018; Madempudi et al., 2019). Further work has demonstrated positive effects in human studies, including in women’s health, metabolism, and mood (Sudha et al., 2011; Ratna Sudha et al., 2012; Majeed et al., 2018; Kazzi et al., 2021).

Currently, the use of natural product-based therapies with antimicrobial properties is gaining attention to introduce new and natural protective agents that do not promote antimicrobial-resistance genes (Martelli and Giacomini, 2018). Among natural preservatives, probiotics are one of the live alternatives due to the synthesis of inhibitory substances such as organic acids, diacetyl, acetoin, hydrogen peroxide and bacteriocins (Konuray and Erginkaya, 2018). Recent studies have emphasized the antimicrobial activities of probiotics as prime importance in the control of pathogenic and food spoilage microorganisms (Fijan, 2023). In a number of studies, the inhibitory effects of *B. coagulans* strains to control pathogenic bacteria have been reported (Donskey et al., 2001; Abada, 2010; Abdhul et al., 2015; De Senna and Lathrop, 2017; Fijan, 2023). Furthermore, *in vitro* studies have investigated *B. coagulans* strains effectiveness in reducing cholesterol, production of lactic acid, bile salt tolerance and antioxidant capacity (Donskey et al., 2001; Abada, 2010; Abdhul et al., 2015; De Senna and Lathrop, 2017).

Whilst, the *in vitro* probiotic potential of a number of *B. coagulans* strains have been well studied, the probiotic potential of *Bacillus coagulans* strain CGI314 has not yet been explored. Additionally, it is well-known that the probiotic potential of microorganisms are strain specific and cannot be generalized among genera, species and strains (Papadimitriou et al., 2015). Herein, the probiotic potential and safety characterization of *B. coagulans* CGI314 was assessed through investigating gastric stress and heat tolerance abilities, cell adhesion, aggregation properties, antibacterial activity, metabolite production, antioxidant capacity, lactate production, cholesterol assimilation, antibiotics susceptibility, hemolytic activity, and cytotoxicity. Moreover, to the best of our knowledge this study will be the first to report the potential of a member of the *B. coagulans* in alleviating hydrogen peroxide (H_2_O_2_)-induced disruption of intestinal-epithelial barrier as measured by tight junction gene expression and western blots, as well as transepithelial electrical resistance (TEER), and dextran permeability methods.

## 2 Materials and method

### 2.1 Bacterial strains and culture conditions

Spore-based probiotic *Bacillus coagulans* CGI314 was provided by Deerland Probiotics and Enzymes (US) and was cultivated at 37°C for 24 h in Tryptic Soy Broth (TSB; Becton, Dickinson and Company, Berkshire, England). Indicator strains employed to determine the antimicrobial activity of *B. coagulans* CGI314 are listed in Table 1, these were either purchased from the American Type Culture Collection (ATCC, Middlesex, United Kingdom) or Deutsche Sammlung von Mikroorganismen und Zellkulturen GmbH (DSMZ, Braunschweig, Germany). These strains were cultivated at 37°C for 24 h, media and other growth conditions used for their cultivation are presented in Table 1. Additionally, other control strains employed for comparative analysis were *Lactobacillus rhamnosus* (GG; ATCC 53103) cultivated at 37°C for 24 h in TSB (Becton, Dickinson and Company, Berkshire, England), and *Lactobacillus fermentum* ME-3 cultivated at 37°C for 24 h in De Man, Rogosa and Sharpe broth (MRS; Merck, Darmstadt, Germany).

**TABLE 1:** List of Indicator strains used in the current study and their culture conditions.

Bacterial species	Strain designation	Media and growth conditions	
*Salmonella enterica*	ATCC13076	TSB	Aerobic, 37°C for 24 h
*Pseudomonas aeruginosa*	DSM3227	TSB	Anerobic, 37°C for 24 h
*Yersinia enterocolitica*	DSM4780	TSB	Aerobic, 37°C for 24 h
*Staphylococcus warneri*	DSM20316	TSB	Aerobic, 37°C for 24 h
*Escherichia coli*	ATCC25922	TSB	Aerobic, 37°C for 24 h
*Staphylococcus epidermidis*	DSM20044	TSB	Aerobic, 37°C for 24 h
*Listeria monocytogenes*	DSM20600	TSB	Aerobic, 37°C for 24 h
*Shigella flexneri*	DSM4782	TSB	Aerobic, 37°C for 24 h
*Candida albicans*	DSM3454	TSB	Aerobic, 37°C for 24 h
*Cutibacterium acnes*	DSM1897	sBHI	Anerobic, 37°C for 24 h
*Staphylococcus pseudintermedius*	DSM21284	TSB	Aerobic, 37°C for 24 h
*Staphylococcus saprophyticus*	DSM20229	TSB	Aerobic, 37°C for 24 h
*Staphylococcus aureus*	DSM1104	TSB	Aerobic, 37°C for 24 h
*Staphylococcus aureus*	RF122	TSB	Aerobic, 37°C for 24 h
*Corynebacterium flavescens*	DSM20296	TSB	Aerobic, 37°C for 24 h
*Acinetobacter baumannii*	DSM30007	TSB	Aerobic, 37°C for 24 h
*Enterococcus faecalis*	DSM20478	TSB	Aerobic, 37°C for 24 h
*Streptococcus agalactiae*	DSM2134	TSB	Aerobic, 37°C for 24 h
*Streptococcus pyogenes*	DSM20565	TSB	Aerobic, 37°C for 24 h
*Gardnerella vaginalis*	DSM4944	sBHI	Anerobic, 37°C for 24 h
*Arcanobacterium haemolyticum*	DSM22856	TSB	Anerobic, 37°C for 24 h
*Campylobacter jejuni*	DSM4688	TSB	Aerobic, 37°C for 24 h
*Campylobacter coli*	DSM4689	sBHI	Anerobic, 37°C for 24 h
*Streptococcus pneumoniae*	DSM24048	sBHI	Anerobic, 37°C for 24 h
*Mobiluncus curtisii*	DSM23059	sBHI	Anerobic, 37°C for 24 h
*Erysipelothrix rhusiopathiae*	DSM5055	sBHI	Aerobic, 37°C for 24 h
*Streptococcus mutans*	DSM20523	TSB	Aerobic, 37°C for 24 h
*Streptococcus sobrinus*	DSM20742	TSB	Aerobic, 37°C for 24 h

### 2.2 Cell culture maintenance

Human colorectal adenocarcinoma cell line HT-29 (ATCC HTB-38™; Sigma-Aldrich, Wicklow, Ireland) and mucous-secreting HT-29-MTX (Sigma-Aldrich, Wicklow, Ireland) were propagated using low glucose DMEM medium supplemented with 10% Fetal Bovine Serum, 2 mM L-glutamine, 100U/ml penicillin, and 100 μg/ml streptomycin in a 5% CO_2_ atmosphere at 37°C. Hepatocellular carcinoma cells line HepG2 [ATCC HB-8065™; American Type Culture Collection (ATCC), Middlesex, UK] cells were routinely maintained in EMEM cell culture media supplemented with 10% fetal bovine serum, 1% non-essential amino acids solution, 100 U/mL penicillin, 100 μg/mL streptomycin and 1% L-Glutamine. Cells were incubated at 37°C under 5% CO_2_ atmosphere.

### 2.3 Stability of *B. coagulans* CGI314 spores during pasteurization

Spore suspension was prepared by adding 50 mg of *B. coagulans* CGI314 spore powders to 20 ml of 1X PBS, pH 7.6 (Sigma-Aldrich). The suspension was mixed using a vortex for 2 min and dispensed into glass test tubes, 5 ml of suspension per tube. Comparator non-spore forming lactic acid bacteria (LAB) strains were used, i.e., *Lactobacillus rhamnosus* GG (ATCC 53103) and *Lactobacillus fermentum* ME-3 cultivated at 37°C for 20 h in 20 mls of TSB and MRS, respectively. After incubation, 5 ml of the culture was dispensed into glass test tubes. All the test tubes were treated at 45°C, 75°C and 90°C in a water bath for 0.5, 1, or 3 min. Following incubation, samples were serially diluted and spread plated on MRS agar. Plates were incubated at 37°C for 24 h- 48 h prior to counting colonies.

### 2.4 Resistance of *B. coagulans* CGI314 spores to simulated gastric and intestinal conditions

The tolerance of *B. coagulans* CGI314 spores to *in vitro* simulated gastric and intestinal conditions was investigated using a modification of the previously described method (Pisano et al., 2014). *L. rhamnosus* GG was used as a control strain as the stability of this strain under simulated gastric and intestinal conditions has been previously described using similar methods (Khokhlova et al., 2023). Briefly, 100 μl of spore suspensions in PBS or 100 μl of *L. rhamnosus* GG suspension in PBS were added to 900 μl of 0.3% pepsin (w/v, Sigma P6887) in NaCl pH 3 and incubated for 2 h at 37°C in a water bath. Following incubation, spores and bacteria were pelleted by centrifugation at 3,000 *g* for 5 min; the pellets were washed two times with 1 ml PBS and resuspended in 100 μl PBS. Thereafter, 900 μl of solution containing 0.1% pancreatin (w/v, Sigma P1750) and 0.3% bile salts (w/v, Sigma cat no. 48305) in PBS (pH 7.5) were added to the spores and bacteria and incubated for another 2 h at 37°C in a water bath. Following 1, 2, 3, and 4 h of incubation, sample aliquots were serially diluted and plated onto TSA plates (*B. coagulans* CGI314 spores) or MRS agar plates (*L. rhamnosus* GG) for colony formation counting.

### 2.5 *B. coagulans* CGI314 adhesion to HT-29 and HT-29-MTX cell lines

HT-29 and HT-29-MTX cells were seeded onto 24-well plates at a density of 5 × 10^5^ cell/well and cultured for 21–28 days to complete maturation. Media was replaced every 2–3 days. Once the required density was attained, 400 μl of full media (low glucose DMEM, 10% Fetal Bovine Serum, 2 mM glutamine) without antibiotics were added to the wells allocated for bacteria; DPBS was aspirated from the wells allocated for spores after the second round of washing. *L. fermentum* was used as a positive control as its ability to adhere to intestinal cell lines is well established (Archer et al., 2018). Cell concentrations for the late logarithmic/stationary phase for bacteria were determined prior to adhesion experiments to ensure the recommended multiplicity of infection (MOI, 1:10–1:100 mammalian cells: bacterial cells) (Letourneau et al., 2011), then 100 μl of bacterial suspension (containing 3.0–8.0 × 10^7^ CFU/ ml) in DMEM were added to HT-29 and HT-29-MTX cells, mixed by a gentle swirl and incubated for 3.5 h in the CO_2_ incubator at 37°C. Control wells not containing mammalian cells were prepared and incubated in parallel in the same way. Upon incubation with bacterial strains HT-29 and HT-29-MTX cells were gently washed four times with 0.5 ml DPBS. After that 100 μl of Trypsin/EDTA solution were added to the wells and incubated for 20 min with gentle shaking (∼100 rpm) at 37°C. 900 μl of PBS were added to each well, contents of the wells were transferred into 1.5 ml Eppendorf tubes with scraping to ensure complete transfer of all material in the wells, and subjected to vigorous shaking for 30 s. Contents of control wells were transferred into microcentrifuge tubes and subjected to one round of shaking in a similar manner. Serial dilutions (plus dilutions of control wells) were prepared in PBS and plated onto MRS agar plates for quantification of *B. coagulans* CGI314 and *L. fermentum* ME-3.

### 2.6 *B. coagulans* auto-aggregation and co-aggregation

Auto-aggregation and co-aggregation analysis were performed in accordance with Walter et al. (2008), Diale et al. (2021) and Zawistowska-Rojek et al. (2022) with the following modifications (Walter et al., 2008; Diale et al., 2021; Zawistowska-Rojek et al., 2022). *B. coagulans* CGI314, *Escherichia coli*, *Enterococcus faecalis* DSM20478, and *Staphylococcus aureus* DSM17091 strains were grown for 20 h in a TSB broth at 37°C. The bacteria were centrifuged at 4,000 × *g* for 15 min, washed twice with 1X PBS and then re-suspended in 1X PBS. 200 ul of cell suspensions were transferred to 96- well plate for quantitative analysis of auto-aggregation by periodically measuring the OD_600_ value using the ThermoFisher Multiskan™ FC Microplate Photometer for up to 24 h at room temperature.

Bacterial suspensions for co-aggregation analysis were prepared with the same method as described for the auto-aggregation test. 100 ul of both *B. coagulans* CGI314 and pathogenic strain suspensions were mixed and then incubated at room temperature without agitation. The absorbance was measured every hour over a 24-h period. The percentage of auto-aggregation and co-aggregation with bacteria was determined as:

$$\frac{A\,b\,s_{0\,h}-A\,b\,s_{24\,h}}{A\,b\,s_{0\,h}}*100$$

### 2.7 *B. coagulans* CGI314 total antioxidant, catalase and GSH activity assay

*Lactobacillus rhamnosus* GG was used as a positive control strain as the antioxidant potential of this strain has been well established (Ren et al., 2019; Yau et al., 2020). Overnight cultures of *B. coagulans* CGI314 and *L. rhamnosus* GG 50 μl were prepared in TSB broth and normalized by number of bacteria (1 × 10^8^ CFU/ ml), then centrifuged at 4,000 *g* for 15 min. Obtained pellets were washed three times with 10 ml phosphate buffered saline (PBS, Sigma-Aldrich) and resuspended in 1 ml PBS. Cell suspensions were transferred to beaded tubes (A29158, Thermofisher). Cells were lysed using BeadBug™ 6 homogenizer at 3,500 rpm, 3 cycles 30 s each, suspensions were kept on ice between cycles for 30 s. Tubes were centrifuged at 9,800 × *g* for 15 min to remove cell debris and the supernatants were transferred to fresh microcentrifuge tubes. Total antioxidant capacity in Trolox equivalents have been measured using total antioxidant capacity assay kit (MAK187, Sigma-Aldrich). The level of catalase and reduced glutathione activity (GSH) in cell lysates have been determined using catalase assay kit (CAK1061, Cohesion Biosciences, UK) and reduced glutathione assay kits (E-BC-K030-S, Elabscience, China) according to the manufacturer’s instructions. Absorbance was measured at wavelengths advised by the manufacture using a reader (Multiskan FC microplate photometer, Thermofisher, Ireland).

### 2.8 *B. coagulans* CGI314 cytoprotective effects against H_2_O_2_-induced cell death

HT-29 and HepG2 cells were seeded at a density of 5 × 10^4^ and 1 × 10^4^ cells per well in 96 well plates, respectively. The plates were incubated at 37°C, 5% CO_2_ for 24 h to allow for cell attachment. The cells were washed twice with 200 μl of DPBS prior to the addition of 100 μl of either prewashed vegetative cells or supernatants of *B. coagulans* CGI314 in HepG2 and HT-29 cell line at 2.4 × 10^5^ CFU/ mL and 6.9 × 10^6^ CFU/mL, respectively. The bacterial cell concentrations were within the recommended MOI [∼ 1:10–1:100 (bacteria: cells)]. In a parallel experiment *B. coagulans* CGI314 cells at 2.4 × 10^5^ and 6.9 × 10^6^ CFU/mL in combination with 2 mM H_2_O_2_ suspended in DMEM without additives were added to HepG2 and HT-29 cells, respectively. All wells were marked up to 100 μl with DMEM without additives. The following day, media containing the treatments were removed and cells were washed with PBS three times. 100 μL of full media and 70 μL of XTT reagent was added to each treated well. The 96 well plate was each incubated at 37°C, 5% CO_2_ for 4 h. To determine the viability of the cells, absorbance reading at 450 nm and 660 nm was recorded using Multiskan FC (Thermofisher scientific, Dublin, Ireland) microplate reader.

### 2.9 *B. coagulans* CGI314 antioxidant capacity in HT-29 and HepG2 cell lines

Antioxidant assays were performed using fluorescent dye—DCF-DA. HT-29 and HepG2 cells were seeded in 100 μL complete media at a density of 5 × 10^4^ and 1 × 10^4^ cells per well in 96 well plates, respectively. The plates were incubated at 37°C, 5% CO_2_ for 24 h to allow for cell attachment. The cells were washed three times with PBS prior to the treatment. The cells were then treated with *B. coagulans* CGI314 supernatants at 2.4 × 10^5^ CFU/mL in HepG2 and at 6.9 × 10^6^ CFU/mL in HT-29 cell line and *B. coagulans* CGI314 cells at 9.74 × 10^4^ CFU/mL in HepG2 and 2.7 × 10^5^ CFU/mL in HT-29 cell line. Ascorbic acid (vitamin C) at 1 mg/mL concentration was used as a positive control for the antioxidant activity. All samples including positive control were prepared in the corresponding cell culture media. The 96 well plates were then returned to the incubator and incubated at 37°C, 5% CO_2_ for 20 h. The medium containing the treatment was removed and cells were washed with PBS prior to staining. The cells were then stained with fluorescent dye at 60 μM DCF-DA concentration in serum-free cell culture media for 50 min. The medium containing the stain was removed and cells were washed with PBS twice prior to addition of insults. Insults at a concentration of 2 mM H_2_O_2_ was prepared in the corresponding serum-free cell culture media. All groups except the control were exposed to the 2 mM H_2_O_2_ insult for 90 min. Fluorescence was measured using a Varioskan lux (Thermofisher scientific, Dublin, Ireland) 485 emission and 538 nm excitation filter set.

### 2.10 Quantitative analysis of amino acids and vitamins in *B. coagulans* CGI314 supernatants in general and milk media

Briefly, *B. coagulans* CGI314 cultivated in TSB at 37°C for 24 h was taken for analysis. Amino acids quantification was carried out using high performance liquid chromatography (HPLC) with fluorescence detection (FLD), using a precolumn derivatization step with 6-aminoquinolyl-*N-hydroxy*succinimidyl carbamate (AQC). Regarding the determination of vitamins, ultra-high performance liquid chromatography (UHPLC) coupled to a simple quadrupole mass detector (MS) was used. The analysis was carried out using a Thermo Scientific Vanquish LC coupled to a Orbitrap Exploris 240 MS, Thermo Fisher Scientific. An electrospray ionization interface was used as ionization source. Analysis was performed in positive and negative ionization mode under polarity switching. The UHPLC was performed using a slightly modified version of the protocol described by Doneanu et al. (2011). Peak areas were extracted using Compound Discoverer 3.3 (Thermo Scientific).

For the metabolite analysis of milk hydrolysates, 1 mL of *B. coagulans* CGI314 (10^8^ CFU mL^–1^) was used to inoculate 50 ml of commercial ultra-high-temperature (UHT) Milk (Indomilk, Semarang, Central Java, Indonesia). The culture was incubated at 37°C for 48 h with shaking under anerobic conditions. After 48 h incubation, 5 ml of the samples were stored at −80°C. Control was uninoculated UHT milk. Mass spectrometry analysis was carried out by MS-Omics (DK-2950 Vedbæk, Denmark) as follows. Samples were acidified using hydrochloride acid, and deuterium-labeled internal standards where added. All samples were analyzed in a randomized order. Analysis was performed using a high polarity column (Zebron™ ZB-FFAP, GC Cap. Column 30 m × 0.25 mm × 0.25 μm) installed in a GC (7890B, Agilent) coupled with a quadrupole detector (5977B, Agilent). The system was controlled by ChemStation (Agilent). Raw data was converted to netCDF format using Chemstation (Agilent), before the data was imported and processed in Matlab R2014b (Mathworks, Inc.) using the PARADISe software as described previously (Johnsen et al., 2017).

### 2.11 Semi-quantitative assays for carbohydrate fermentation and hydrolytic activities

The ability of *B. coagulans* CGI314 to use different carbon sources was investigated with API 50CH strips (BioMérieux, Hampshire, UK), while hydrolytic activities were determined using the API-ZYM kit system (BioMérieux, Hampshire, UK) according to the instructions provided by the manufacturer.

### 2.12 Cholesterol reduction assay

*Lactobacillus rhamnosus* GG is used as a control strain as cholesterol lowering abilities of this strain have been reported previously (Kim et al., 2016). *B. coagulans* CGI314 and *L. rhamnosus* GG were cultivated in TSB at 37°C, 170 RPM for 20 h, after which time 100 μl of the bacterial cultures were transferred to 10 ml TSB supplemented with 6 mM of water-soluble cholesterol in 50 ml conical flasks. The cultures were incubated for up to 72 h at 37°C, 170 RPM. After a predetermined time, interval (0, 4, 8, 24, 32, 48, 72 h), the cultures were withdrawn and centrifuged (4,000 × *g*, at 4°C for 10 min). After centrifugation, 500 μl of culture supernatant was used to determine cholesterol content using Total cholesterol microplate assay kit (CAK1116) according to manufacturer’s instruction.

### 2.13 Production of lactate

*Bacillus coagulans* CGI314, *L. rhamnosus* GG, and *L. fermentum* ME-3 were cultivated in 10 ml TSB media and MRS media at 37°C, 170 RPM for 24 h. The following day, 100 μl of the overnight cultures were transferred to 10 ml of TSB and MRS media in 50 ml conical flasks. The cultures were incubated for 48 h at 37°C, 170 RPM. Ten milliliters of the probiotic strains were transferred into new 15 ml falcon tubes. The cultures were serially diluted to 10^–6^ and plated in TSA/MRS agar plates. The plates were incubated at 37° for 48 h. The remaining undiluted cultures were stored at 4°C until required. After incubation, counts were recorded, and all 3 strains were normalized to 1 x 10^8^ CFU/ ml using 1X PBS. The tubes were centrifuged at 9,800 × *G* for 15 min and supernatants were stored at −20°C or assayed directly. In a parallel experiment, pellets were washed in 10 ml 1X PBS. Washing and centrifugation of the pellet with 10 ml 1X PBS was repeated twice. The pellets were resuspended in 1 ml lactate assay buffer (from the assay kit; CAK1177) and vortexed vigorously. The suspensions were transferred to fresh beaded tubes. *B. coagulans* CGI314, *L. rhamnosus* GG and *L. fermentum* ME-3 were homogenized at 3,500 rpm for 30 s using the Beadbug homogenizer. The tubes were placed in ice for 30 s between cycles. The process was repeated three times. The homogenized samples were centrifuged at 9,800 × *G* for 15 min. The supernatants (cell lysate or intracellular matrix) were recovered and stored at −20°C or assayed directly. Lactate assay buffer was set as negative control. Cell free suspension and cell lysates were tested for lactate content using lactate assay kit (CAK1177) according to the manufacturer’s manual. Absorbance reading at 450 nm was recorded using Multiskan FC (Thermofisher scientific, Dublin, Ireland) microplate reader.

### 2.14 Antimicrobial activity of *B. coagulans* CGI314

A modification of a double layered agar method was used for the determination of antimicrobial activity of *B. coagulans* CGI314 against indicator strains summarized in Table 1 (Stachurska et al., 2021). MRS agar was used as a base layer and was spotted with 30 μl of stationary phase CGI314 culture (approximately 10^8^ CFU/mL). The spots were then left to dry, and the plates were incubated at 37°C for 20 h. Five milliliters of molten TSA or sBHI (0.3–0.5% agar) inoculated with 100 μl of 1:100 diluted stationary phase indicator strain suspension (approximately 10^9^ CFU / mL) were used to overlay the plates. The plates were incubated at 37°C for 24 h and the inhibition zones against the test strains were observed.

### 2.15 Anti-inflammatory activity assay

HT-29 cells were seeded onto 24-well plates at a density of 5 × 10^5^ cell/well. After 24 h of incubation cells were washed twice with 0.5 ml DPBS prior to adding 0.4 ml of full cell culture media without antibiotics to the wells allotted for pretreatment with bacteria. One hundred microliters of washed viable *B. coagulans* CGI314 (2 × 10^8^ CFU/mL) or heat treated to 121°C for 15 min cells were added to the corresponding wells. 500 μl of full cell culture medium were added to the control wells. After 20 h of incubation (CO_2_ atmosphere at 37°C), HT-29 cells were washed twice with 0.5 ml of DPBS and 0.5 ml of full cell culture medium containing antibiotics were added to the wells. Lipopolysaccharides from *E. coli* O111:B4 (LPS, Sigma L4391) are known increase the levels of several inflammatory markers (Li et al., 2020) and therefore were added to HT-29 cells pretreated with viable, heat-treated bacteria and control wells to a final concentration of 10 ng/ml. Three and a half hours after adding LPS, cell culture supernatants were removed, and HT-29 cells were lysed in the wells by adding 300 μl of lysis buffer supplied with Monarch Total RNA Miniprep Kit (NEB MA, USA) and the total RNA was extracted from cell lysates using Monarch Total RNA Miniprep Kit (NEB) according to manufacturer’s instructions. Fifty microliters of nuclease-free water were taken to elute RNA. Qubit™ RNA broad range kit (Thermofisher scientific) was used to quantify RNA after isolation. Up to one microgram of total RNA was taken to set up reverse transcription reactions using OneStepPlus™ system (Applied Biosystems™). Real-time PCR reactions were set up using Luna^®^ Universal qPCR Master Mix (NEB) and appropriate primer pairs (see Supplementary Table 6) at a concentration of 200 nM using 1 μl of generated cDNA per 9 μl of master mix. The reactions were performed in duplicates using the following program: initial denaturation 95°C 5 min, denaturation 94°C 20 s, annealing 60°C 20 s, extension 72°C 20 s (40 cycles). The specificity of reaction products was confirmed by melting temperature analysis (from 70°C to 95°C in 0.5°C/15 s increments). Quantification of target transcripts was done using gapdh as a normalizing house-keeping gene (Supplementary Table 6).

### 2.16 Tight junction protein expression-induction by *B. coagulans* CGI314

HT-29-MTX was propagated using DMEM medium supplemented with 10% Fetal Bovine Serum, 100U/ml penicillin, and 100 μg/ml streptomycin in a 5% CO_2_ atmosphere at 37°C. Cells were seeded were seeded onto transwell filter inserts (Falcon^®^ cell culture inserts, PET, pore size 1 μm; 0.3 cm^2^) at a density of 1 × 10^5^ cell/transwell and cultured for 25–28 days to complete differentiation. The volume of medium was 0.2 ml in the apical compartment and 0.5 ml in the basolateral compartment. Medium was replaced every 2 days. Epithelial tightness was assessed via measurement of the transepithelial electrical resistance (TEER) using a Millicell^®^ ERS-2 device (Millipore). *B. coagulans* CGI314 was propagated using TSB medium. 10 mL of liquid medium were inoculated with 100 μl of overnight cultures and incubated for 16–20 h with vigorous shaking ∼170 rpm. Cultures were transferred into 50 ml falcon tubes and centrifuged for 10 min at 2,500 *g*. Supernatants were removed and bacteria were washed once with 10 ml serum free additives free DMEM. 10 mL of serum free additives free DMEM were added to the pellets. Transwells and wells of the companion plates allocated for bacterial treatment were carefully washed twice with DPBS. 100 μl of full cell culture medium without antibiotics and 100 μl of bacterial suspension, containing 6.0–8.0 × 10^8^ CFU/ml of *B. coagulans* CGI314, prepared as described above were added to the apical compartment. The bacterial cell concentrations were within the recommended MOI [∼ 1:10–1:100 (bacteria: cells)], and 200 μl of full cell culture medium were added to the apical compartment of control transwells. Inserts were incubated for 20 h in a 5% CO_2_ atmosphere at 37°C.

Before and after incubation, TEER was evaluated using a Volt/Ohm meter. Transwell inserts were carefully washed three times with 200 μl of DPBS. After that epithelial cells were lysed in transwells either with 300 μl of lysis buffer supplied with RNA extraction kit or with 150 μl of lysis buffer containing 0.15 M NaCl, 5 mM EDTA, 1% SDS, 10 mM Tris–HCl, pH 8.0. Total RNA was extracted from cell lysates using Monarch Total RNA Miniprep Kit (NEB) according to manufacturer’s instructions. Fifty microliters of nuclease-free water were taken to elute RNA. Qubit™ RNA broad range kit was used to quantify RNA after isolation. A maximum of one microgram of total RNA was taken to set up reverse transcription reactions using Luna script RT Supermix kit (NEB). Real-time PCR reactions were set up using Luna^®^ Universal qPCR Master Mix (NEB) and appropriate primer pairs (see Supplementary Table 6) at a concentration of 200 nM using 2 μl of generated cDNA per 8 μl of qPCR master mix. The reactions were performed in duplicate using the following program: initial denaturation 95°C 5 min, denaturation 94°C 20 s, annealing 60°C 20 s, extension 72°C 20 s (40 cycles). The specificity of reaction products was confirmed by melting temperature analysis (from 70°C to 95°C in 0.5°C/15 s increments). Quantification of target transcripts was done using gapdh as a normalizing house-keeping gene (Supplementary Table 6).

Additionally, cell lysates for Western blotting were mixed with 37.5 μl of 5x Laemmli sample buffer and incubated at 100°C for 5 min. Samples (10–20 μl) were then separated using NuPAGE 4–12% Bis-Tris gels and then transferred onto 0.2 μm nitrocellulose membrane using iBlot™ 2 gel transfer device (Invitrogen/ThermoFisher Scientific) and iBlot™ 2 NC Mini transfer stacks (Invitrogen/ThermoFisher Scientific). The pre-set P0 template (20 V for 1 min, 23 V for 4 min, 25 V for 2 min) was used for detection of occludin and β-actin, the P3 template (20 V for 7 min) was used for detection of claudin 1, for detection of ZO-1 proteins were transferred at 20 V for 10 min. The membranes were blocked in and stained using rabbit antibodies against β-actin, occludin, claudin 1, ZO-1, and HPR-conjugated goat anti-rabbit secondary antibodies (ABclonal) using iBind™ device (Invitrogen/ThermoFisher Scientific). IBind™ Solution kit was used for blocking the membranes and diluting the antibodies in accordance with manufacturer’s instructions. The dilutions used and the catalogue numbers of the antibodies can be found in Supplementary Table 7. Blots were developed using SuperSignal™ West Pico PLUS Chemiluminescent Substrate (Thermo Scientific) according to the manufacturer’s instructions and visualized using iBright™ imaging system (Invitrogen/ThermoFisher Scientific).

### 2.17 Measuring H_2_O_2_-induced disruption of intestinal-epithelial barrier using TEER and dextran permeability

HT-29-MTX was propagated using full media in a 5% CO_2_ atmosphere at 37°C. Cells were seeded onto transwell filter inserts (cellQART^®^, PET, pore size 0.4 μm; 0.3 cm^2^) at a density of 1 × 10^5^ cell/transwell and cultured for 25–28 days to complete differentiation. The volume of medium was 0.3 ml in the apical compartment and 0.7 ml in the basolateral compartment. Medium was replaced every 2 days. Epithelial tightness was assessed via measurement of the transepithelial electrical resistance (TEER) using a Millicell^®^ ERS-2 device (Millipore). *B. coagulans* CGI314 was propagated using TSB medium. Ten milliliters of liquid medium were inoculated with 100 μl of overnight cultures and incubated for 16–20 h with vigorous shaking ∼170 rpm. Cultures were transferred into 50 ml falcon tubes and centrifuged for 10 min at 2,500 *g*. Supernatants were removed and bacteria were washed once with 10 ml serum free additives free DMEM. Ten milliliters of serum free additives free DMEM were added to the pellets. To obtain cell free supernatants bacterial preparations in DMEM were further cultivated for 18 h under conditions indicated above. After that, cultures were centrifuged for 10 min at 4,000 *g*. Pellets were discarded and spent supernatants were filter-sterilized using syringe-mounted filters with 0.22 μm pore diameter. 1N NaOH was added to cell-free supernatant to bring pH to ∼7.5. On the day of the experiment the inserts and transwells allocated for bacterial treatment were washed twice with DPBS. One hundred and fifty microliters of full cell culture medium without antibiotics and 150 μl of bacterial suspension or cell free supernatants prepared as described above were added to the apical compartment. Three hundred microliters of full cell culture medium were added to the apical compartment of control wells. Cells were incubated for 20 h in a 5% CO_2_ atmosphere at 37°C. Upon incubation all transwells (including control wells) were washed three times with DPBS. Serum free DMEM with glutamine and antibiotics were added to the wells, followed by H_2_O_2_ added to both apical and basolateral compartments to a final concentration of 1 mM except for negative control wells. Transwells were incubated for 48 h, H_2_O_2_ was replenished after 24 h. TEER was measured using a Volt/Ohm meter before adding H_2_O_2_ and after 48 h period of incubation. The transwells were washed three times with HBSS, same buffer was taken to fill the transwells, and 4 kDa fluorescein isothiocyanate (FITC)-labeled dextran was added to the apical compartment to a final concentration of 1 mg/ml. After 4 h, the contents of the basolateral compartments were collected, and fluorescence was measured in triplicate in a Varioskan LUX Multimode Microplate Reader (ThermoFisher Scientific). The amount of FITC-dextran concentration in the basal compartment was determined from a standard curve obtained for FITC-dextran ranging from 0.8 to 25 μg/ml.

### 2.18 Statistical analysis

All data was analyzed using Prism 9 (GraphPad Software, CA, USA). Normal distribution was determined using Shapiro-Wilk test. Samples were tested for significance using unpaired t test or one-way ANOVA with Dunnett’s *post-hoc*. When samples did not follow normal distribution, a Mann-Whitney U test or Kruskal-Wallis with Dunn’s *post-hoc* were performed.

## 3 Results

### 3.1 *In vitro* safety evaluation of *B. coagulans* CGI314

#### 3.1.1 Hemolytic activity

**FIGURE 1:**
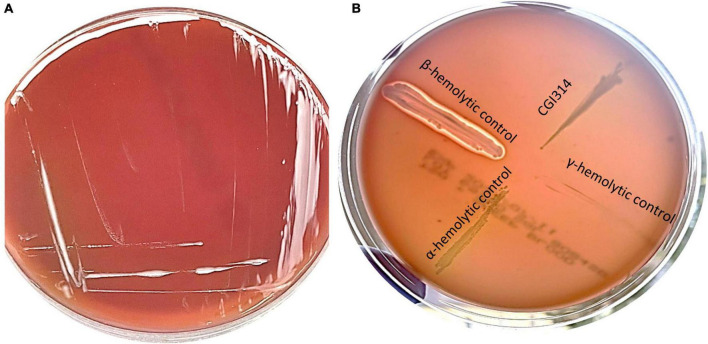
**(A)** Streak plate of *Bacillus coagulans* CGI314 on a sheep blood agar plate, no hemolysis (gamma) occurred on SBA after 24 h incubation at 37°C. **(B)** Control strains of bacteria were used to demonstrate hemolysis patters on SBA. Beta-hemolysis is seen around *Staphylococcus aureus* ATCC 25923 growth, alpha-hemolysis around *Streptococcus pneumoniae* ATCC 25923 and gamma-hemolysis (none) around *E. coli* ATCC 25922 and *B. coagulans* CGI314.

Hemolytic activity of CGI314 strain was evaluated on sheep blood agar plate. CGI314 showed γ hemolytic, i.e., negative, or no hemolytic activity (Figure 1).

#### 3.1.2 Cytotoxicity

**FIGURE 2:**
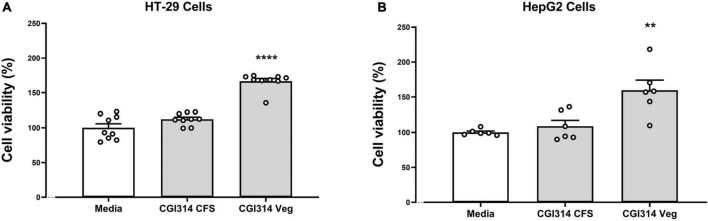
*Bacillus coagulans* CGI314 CFS and vegetative cells were non cytotoxic in **(A)** intestinal epithelial HT-29 cells (*n* = 9) [*F*(2,24) = 66.26, *P* < 0.0001] and **(B)** hepatocellular carcinoma HepG2 cells (*n* = 6) [*F*(2,15) = 10.95, *P* = 0.0012]. Data represents percentage of XTT dye conversion after 20 h of coincubation with *B. coagulans* CGI1314 compared with untreated cells (Media). ****Represents *post hoc p*-value is < 0.0001, **represents *post hoc p*-value is < 0.01.

In this experiment, an XTT (2,3-Bis-(2-Methoxy-4-Nitro-5-Sulfophenyl)-2H-Tetrazolium-5-Carboxanilide) assay was used to observe the effect of CGI314 on HT-29 and HepG2 cell viability. The results showed that in both cell lines, CGI314 cell-free supernatants (CFS) and vegetative cells did not significantly damage cells after 20 h of incubation (Figure 2).

#### 3.1.3 Antibiotics susceptibility

The antibiotic susceptibility of *B. coagulans* CGI314 was evaluated by using antimicrobial agents prescribed by EFSA for the genus *Bacillus* (Rychen et al., 2018). The Minimum Inhibitory Concentration (MIC) values of antibiotics against CGI314 for all of the tested antibiotics (vancomycin, gentamicin, kanamycin, streptomycin, clindamycin, erythromycin, tetracycline and chloramphenicol) showed a MIC range of 0.125–2 mg/L, indicating its susceptibility against antibiotics, meeting the requirements of EFSA (Supplementary Table 1).

### 3.2 *In vitro* probiotic evaluation of *B. coagulans* CGI314

#### 3.2.1 *B. coagulans* CGI314 spores displays strong ability to survive harsh temperatures

**FIGURE 3:**
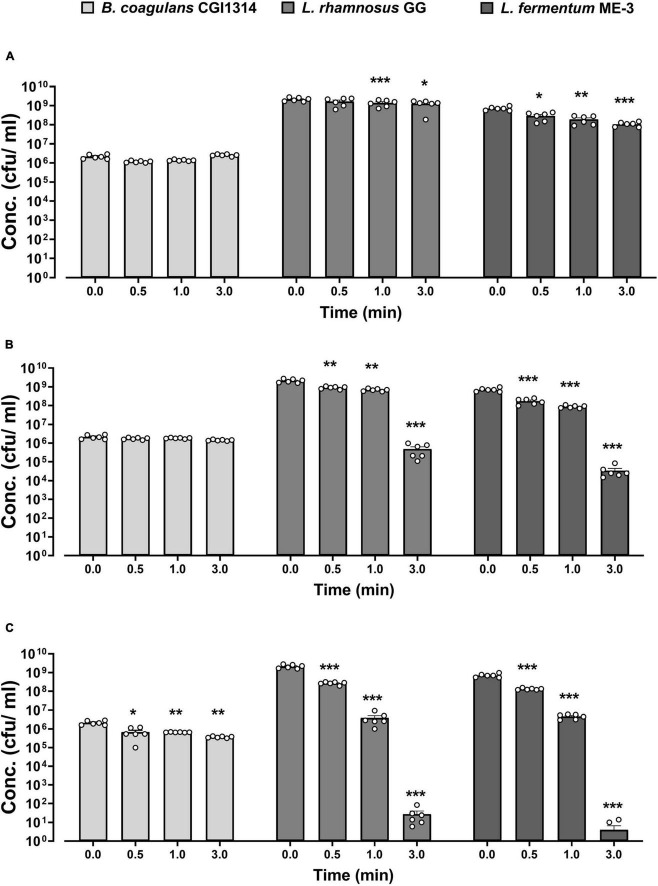
*Bacillus coagulans* CGI314 spores demonstrate superior thermostability over *L. rhamnosus* GG (ATCC 53103) and *L. fermentum* ME-3 at **(A)** 45, **(B)** 75, and **(C)** 90°C over different time intervals. The values are expressed in CFU/ml ± SEM (*n* = 6). Statistical analysis was performed using repeated measures one-way ANOVA, with Dunnett’s *post-hoc* test within each group. There was a significant effect of time for 45°C **(A)** [*F*(2.013,30.20) = 16.02, *p* < 0.001], 75°C **(B)** [*F*(1.146,17.18) = 147.4, *p* < 0.001] and 90°C **(C)** [*F*(1.014,15.20) = 173.9, *p* < 0.001]. ***, **, and * represent *post-hoc p*-values of < 0.001, < 0.01 and < 0.05, respectively, against each individual group’s control concentration at timepoint 0.

The temperature stability of CGI314 spores was assessed at various conditions (45, 75, and 90°C) at three different time points (0.5, 1, and 3 min) in PBS and was compared with that of the commercial *L. rhamnosus* GG (ATCC 15103) and *L. fermentum* ME-3. The summarized results are shown in Figure 3. No change in CGI314 spores viability has been detected at 45°C and 75°C at all three time points. Comparatively, a significant drop in bacterial counts for *L. rhamnosus* and *L. fermentum* were detected at these temperatures. However, after treatment at 90°C we recorded a reduction in CGI314 spores counts from 2.15 × 10^6^ to 3.68 × 10^5^ CFU/mL. Overall, *B. coagulans* CGI314 spores demonstrate strong stability at pasteurization conditions.

#### 3.2.2 *B. coagulans* CGI314 spores show resistance to gastric and intestinal conditions

**FIGURE 4:**
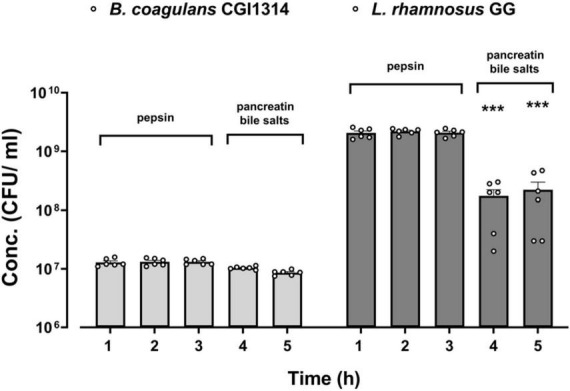
*Bacillus coagulans* CGI314 spores demonstrate a superior resilience to simulated *in vitro* gastric and small intestinal digestion conditions over *L. rhamnosus* GG. The values are expressed in CFU/ml ± SEM (*n* = 6) of two independent experiments performed in 3 technical replicates. Statistical analysis was performed using repeated measures one-way ANOVA, with Dunnett’s *post-hoc* test within each group. There was a significant effect of time [*F*(1.392,13.92) = 103.8, *p* < 0.001]. ***Represents a significant difference with *p* < 0.001, as determined by Dunnett’s *post-hoc* test.

The ability of *B. coagulans* CGI314 spores to survive during an *in vitro* simulated digestion process was compared to that of the commercial *L. rhamnosus* GG (ATCC 15103) strain, as its ability to survive in these conditions using this method has been previously reported (Khokhlova et al., 2023). After exposure to gastric and small intestinal conditions there was no significant decrease in *B. coagulans* CGI314 spores counts, as shown in Figure 4. Whereas, after 2 h of exposure to an intestinal stage simulation (0.1% pancreatin, 0.3% bile salts) there was a significant decrease in viable bacterial counts for *L. rhamnosus* GG (Figure 4). These findings suggest a superior resilience of *B. coagulans* CGI314 spores to survive the transit through the upper digestive tract in comparison to the conventional *Lactobacillus* probiotic strains.

#### 3.2.3 *B. coagulans* CGI314 adhere to mucous secreting intestinal epithelial cell line

**FIGURE 5:**
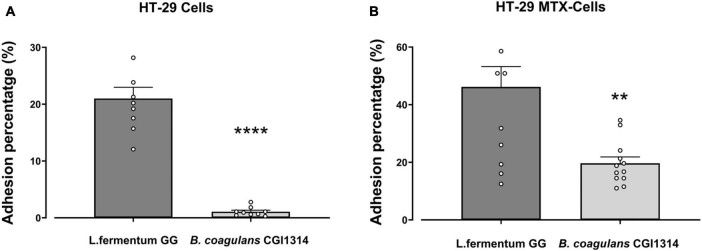
*Bacillus coagulans* CGI314 spores had lower percentage adherence in comparison to *L. fermentum* (control) in **(A)** HT-29 cells (*n* = 9) [*t*_16_ = 9.973, *p* < 0.0001] and **(B)** mucous secreting HT-29 cells [*t*_22_ = 3.589, *p* = 0.0016] (*n* = 12) as determined by 2-tailed *t*-test. **** and ** represent a significant difference with *p* < 0.0001 and *p* < 0.01, respectively.

The adhesion ability of *B. coagulans* CGI314 and *L. fermentum* GG (control) was studied in human colorectal adenocarcinoma cell lines HT-29 and mucus-secreting HT-29-MTX. *L. fermentum* was used as a positive control as its ability to adhere to intestinal cell lines is well established (Archer et al., 2018). *B. coagulans* CGI314 spores displayed low adherence to the non-mucous secreting cell line HT-29, and moderate adherence to the mucous-secreting HT-29-MTX cell line. *B. coagulans* CGI314 spores demonstrated lower adhesion affinity compared with *L. fermentum* GG in both cell lines (Figure 5).

#### 3.2.4 *B. coagulans* CGI314 auto-aggregation and co-aggregation capacity

The level of auto-aggregation of *B. coagulans* CGI314 and co-aggregation between *B. coagulans* CGI314 and pathogenic bacteria (Supplementary Figure 1) over 24 h of incubation was investigated in this study. *B. coagulans* CGI314 displayed moderate auto-aggregation ability with a value of 25.8% (Supplementary Table 2). All of the other strains tested in this study displayed a lower ability to auto-aggregate, at the level from 0.1% for *E. coli* ATCC25922 to 21.42% for *S. aureus* DSM17091. *B. coagulans* CGI314 was able to co-aggregate with all pathogenic strains tested. The highest co-aggregation was 21.85% with *E. faecalis* DSM20478, and the lowest was at 10.5% with *E. coli* ATCC25922 (Supplementary Figure 1; Supplementary Table 2).

#### 3.2.5 *B. coagulans* CGI314 antioxidant capacity

**FIGURE 6:**
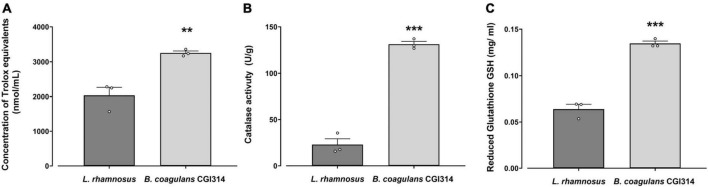
*Bacillus coagulans* CGI314 (1 × 10^8^ CFU / mL) demonstrated superior antioxidant capacity over *L. rhamnosus* GG in panel **(A)**. Total antioxidant capacity in Trolox equivalents (nmol/g) [*t*5 = 5.104, ** represents a significant difference with *p* = 0.007] **(B)** catalase activity (in U/g) [*t*5 = 15.49, *** represents a significant difference with *p* = 0.0001], and **(C)** reduced glutathione GSH (in mg/ml) activity assays [*t*5 = 12.02, *** represents a significant difference with *p* = 0.0003] as determined by Student’s *t*-test.

Oxidants, such as reactive oxygen species (ROS) and reactive nitrogen species (RNS), can generate free radicals that can cause severe oxidative damage to cellular lipids, membranes, proteins, and DNA (Mazhar et al., 2023; Simon et al., 2023b). Antioxidants can scavenge these free radicals and prevent cellular oxidative stress by enzymatic and non-enzymatic mechanisms (Valko et al., 2007). In this study, total antioxidant capacity in addition to catalase and reduced glutathione (GSH) assays were used to characterize *B. coagulans* CGI314 antioxidant potential. *L. rhamnosus* GG was used as a comparator strain as its antioxidant capacity has been reported previously using both *in vitro* and *in vivo* models (Yau et al., 2020; Ma et al., 2022; Khokhlova et al., 2023; Simon et al., 2023a). While, both *B. coagulans* CGI314 and *L. rhamnosus* GG demonstrated antioxidant activity when measured by these assays, *B. coagulans* CGI314 demonstrated superior antioxidant capacity in all assays performed (Figure 6).

#### 3.2.6 *B. coagulans* CGI314 cytoprotective and antioxidant capacity in HT-29 and HepG2 cell lines

**FIGURE 7:**
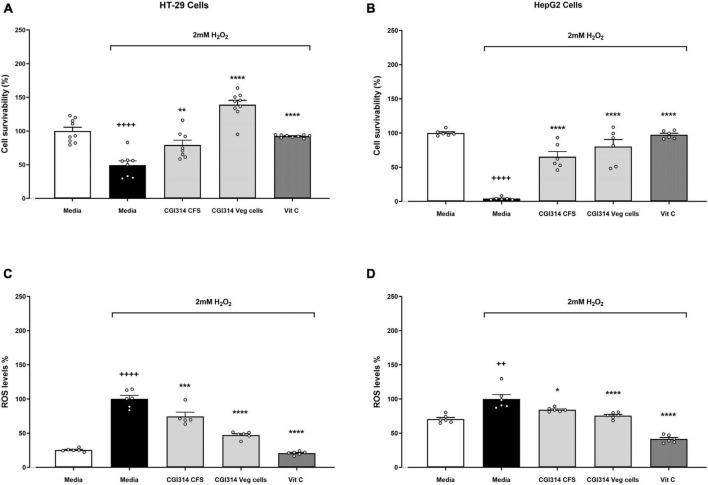
*Bacillus coagulans* CGI314 vegetative cells and cell-free supernatant (CFS) significantly attenuated 2mM H_2_O_2_-induced cell damage in **(A)** HT-29 (*n* = 9) and **(B)** HepG2 (*n* = 6) cell lines; and 2mM H_2_O_2_-induced ROS production in **(C)** HT-29 (*n* = 6) and **(D)** HepG2 (*n* = 6) cell lines. H_2_O_2_ treatment survivability in **(A)** HT-29 cells [*t*15 = 5.929, ^++++^*p* < 0.0001] and **(B)** HepG2 cells [*t*10 = 48.76, ^++++^*p* < 0.0001]; and increased ROS production in **(C)** HT-29 cells [*t*10 = 14.49, ^++++^*p* < 0.0001] and **(D)** [*t*10 = 4.257, ^++^*p* = 0.0017]. One-way ANOVA followed by Dunnett’s *post-hoc* test for all H_2_O_2_-treated groups determined a treatment effect for cell survivability in HT-29 cells **(A)** *F*(3,30) = 43.59, *p* < 0.0001 and **(B)** HepG2 cells *F*(3,20) = 38.62, *p* < 0.000; as well as a treatment effect for the reduction of ROS production in **(C)** HT-29 cells *F*(3,19) = 77.79, *p* < 0.0001 and **(D)** HepG2 cells *F*(3,20) = 47.27, *p* < 0.0001. ****, ***, ** and * represent *post-hoc p*-values of < 0.0001, < 0.001, < 0.01 and < 0.05, respectively, against the media group that was treated with H_2_O_2_.

After determining *B. coagulans* CGI314 had strong antioxidant enzyme activity, the strain was subjected to further analysis by evaluating its cytoprotective capacity to protect against oxidative stress on intestinal and liver cell lines induced by H_2_O_2_. In both cell lines, *B. coagulans* CGI314 vegetative cells and CFS in combination with 2mM H_2_O_2_ offered cytoprotective effects (Figures 7A, B). Additionally, in both cell lines *B. coagulans* CGI314 vegetative cells and CFS displayed a statistically significant reduction in ROS induced by 2mM H_2_O_2_. The cytoprotective and ROS reduction activities of *B. coagulans* CGI314 were comparable to that of the positive control, Vitamin C at 1 mg/ ml (Figures 7C, D).

#### 3.2.7 Amino acids and vitamins in *B. coagulans* CGI314 supernatant

**FIGURE 8:**
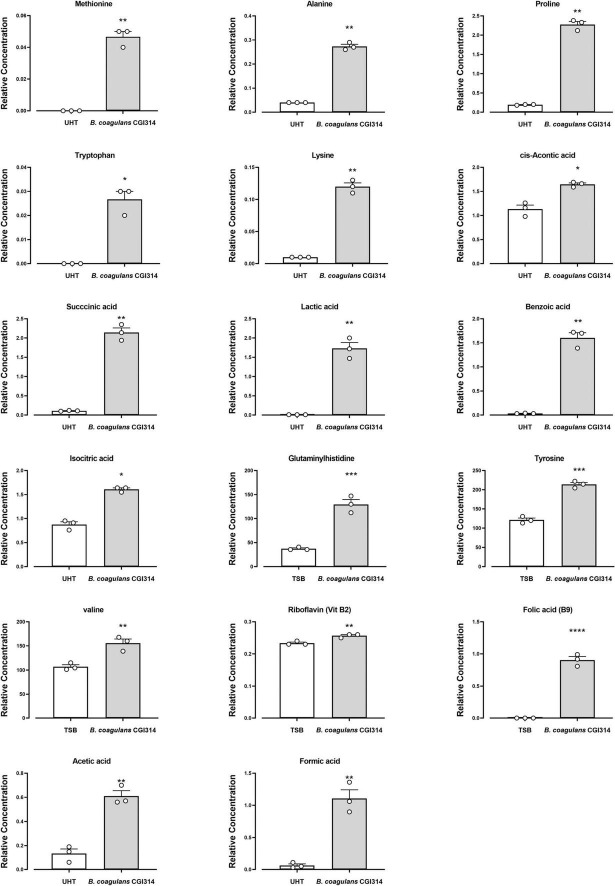
*Bacillus coagulans* CGI314 significantly increase three amino acids and two vitamins in TSB media as determined using HPLC-FLD and UHPLC-MS, correspondingly. Ten amino acids and 2 SCFA significantly increased in *B. coagulans* CGI314 fermented UHT milk, determined using GC-MS. The results show average concentration (*n* = 3) ± standard error. Significant difference observed between control (TSB and UHT milk) and *B. coagulans* CGI1314: *p < 0.05, **p < 0.01, ***p < 0.001, and ****p < 0.0001. The results were statistically analyzed using the student’s *t*-test in GraphPad Prism v.9.

Furthermore, we performed an exploratory study to assess the metabolomic profile of TSB and UHT milk fermented with *B. coagulans* CGI314. In TSB a total of 20 amino acids and 9 vitamins were identified, out of which 3 amino acids (glutamyl histidine, valine and tyrosine) and 2 water soluble vitamins (B2 and B9) were significantly altered by the presence of *B. coagulans* CGI314 (Figure 8). Additionally, in UHT milk a total of 40 amino acids were identified, out of which 10 were found to be significantly increased by *B. coagulans* CGI314, these were methionine, alanine, proline, tryptophan, lysine, *cis-*aconitic acid, succinic acid, lactic acid, benzoic acid, and isocitric acid (Figure 8). This data suggests the potential capacity of *B. coagulans* CGI314 to synthesize the above-mentioned amino acids and vitamins from the breakdown of complex molecules or by employing *de novo* synthesis. In addition to amino acid and vitamins, a total of nine short chain fatty acid (SCFA) compounds were identified in the CGI314 fermented UHT milk, of which two (acetic acid and formic acid) were found to be statistically significant increased by CGI314 (Figure 8; Supplementary Table 3).

#### 3.2.8 Carbohydrate fermentation and enzyme profile of *B. coagulans* CGI314

The carbohydrate assimilation pattern determined using the API 50 CH assay kit is shown in Supplementary Table 4. *B. coagulans* CGI314 was positive for metabolism of 22 carbohydrates out of 49 tested using commercial API 50 CH strip which includes; L-arabinose, D-ribose, D-xylose, D-galactose, D-glucose, D-rhamnose, D-fructose, D-mannose, D-mannitol, D-sorbitol, Methyl-ad-glucopyranoside, N-Acetylglucosamine, Amygdalin, Arbutin, Esculin ferric citrate, Salicin, D-cellobiose, D-maltose, D-melibiose, D-trehalose, Amidon, Gentibiose, D-arabitol, and Potassium gluconate. Enzymatic profile of *B. coagulans* CGI314 detected using the API ZYM kit is presented in Supplementary Table 5. *B. coagulans* CGI314 was positive for esterase (esterase-lipase C8 and C4), peptidase (leucine arylamidase, valine arylamidase, and cystine arylamidase), phosphatase (acid, alkaline phosphatase and phosphohydrolase), and glycosidase (α -galactosidase, α -glucosidase, β-galactosidase, and β-glucosidase) activity.

#### 3.2.9 Cholesterol-reducing ability of *B. coagulans* CGI314

**FIGURE 9:**
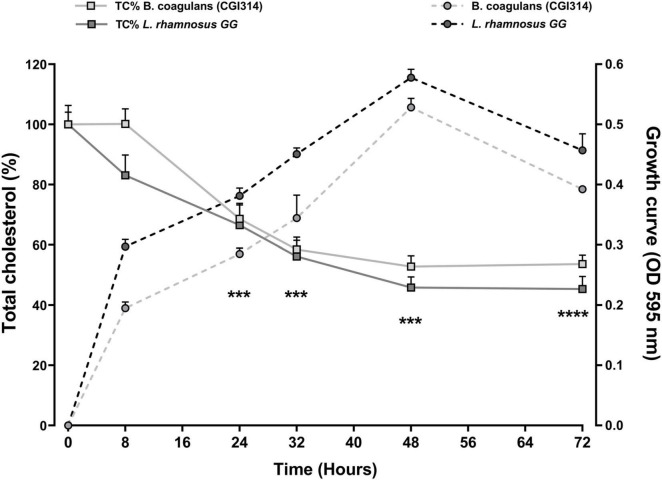
*Bacillus coagulans* CGI314 and *L. rhamnosus* GG growth and significant removal of total cholesterol (Tc%) in TSB media supplemented with 6mM water-soluble cholesterol for 72 h at 37°C. *B. coagulans* CGI314 and *L. rhamnosus* GG significantly reduced cholesterol from timepoint 24 h onward as compared with starting concentration using 2-way ANOVA followed by Dunnett’s multiple comparison test [*F*(1.430,5.720) = 72.22, ****p = 0.0001]. There were no between group effects. Each value represents the mean ± SEM (*n* = 3). *** represents significant differences as compared to both group’s respective starting cholesterol concentration *p* < 0.001.

The removal of cholesterol by *B. coagulans* CGI314 grown in TSB broth supplemented with 6mM water-soluble cholesterol for 72 h at 37°C presented in Figure 9. *L. rhamnosus* GG was used as a comparator strain as cholesterol lowering abilities of this strain have been reported previously (Kim et al., 2016). Results revealed that *B. coagulans* CGI314 can remove 50% of total cholesterol from its growth medium after 24 h of incubation. According to two-way ANOVA analysis, there was a significant cholesterol reduction at 24 h upto 48 h of growth, suggesting that *B. coagulans* CGI314 was more effective at removing cholesterol in the growth phase (***p < 0.001). Overall, no significant difference in the removal of total cholesterol from the TSB media was observed between *B. coagulans* CGI314 and *L. rhamnosus* GG over the 72 h of incubation (Figure 9).

#### 3.2.10 Lactate production by *B. coagulans* CGI314

**FIGURE 10:**
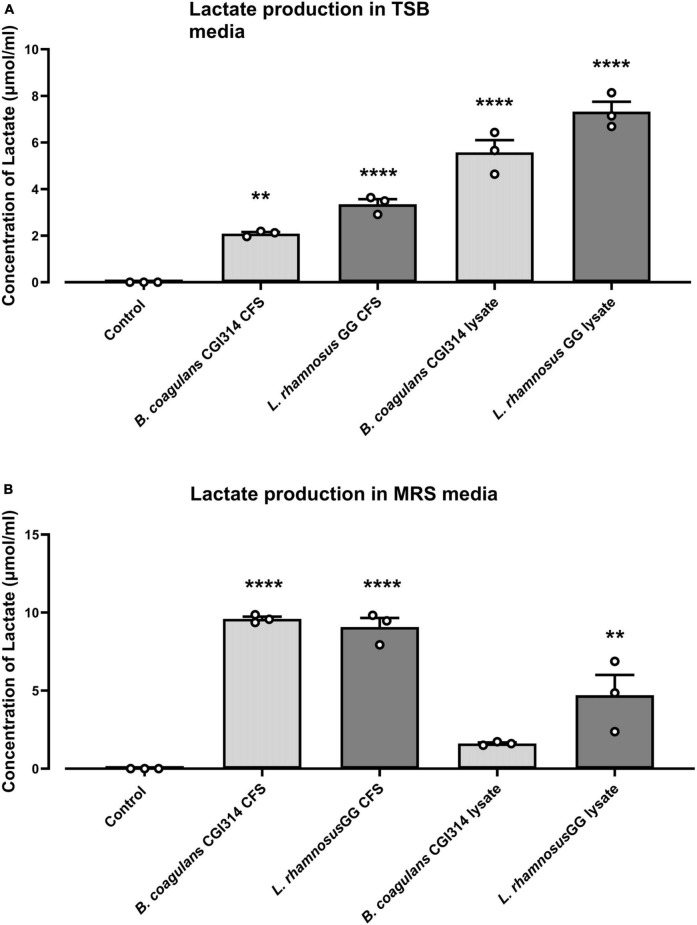
*Bacillus coagulans* CGI314 and *L. rhamnosus* GG significantly increased lactate production in **(A)** TSB and **(B)** MRS media (*n* = 3) ± standard error. One-way ANOVA followed by Dunnett’s multiple comparison test determined significantly increased lactate production on **(A)** TSB [*F*(4,10) = 81.66, *P* < 0.0001] and **(B)** MRS media [*F*(4,10) = 45.24, *P* < 0.0001]. **** and ** represent a *post-hoc* significant difference with *p* < 0.0001 and *p* < 0.01, respectively.

Lactate production by *B. coagulans* CGI314 and *L. rhamnosus* GG grown in TSB and MRS media was quantified using lactate microplate assay kit. Both, *B. coagulans* CGI314 and *L. rhamnosus* GG demonstrated strong lactate activity. In both, MRS and TSB media the concentration of lactate production in CFS and cell lysates was similar for *B. coagulans* CGI314 and *L. rhamnosus* GG (Figure 10) except for CGI314 CFS in TSB (Figure 10A). Overall, results from this assay revealed CGI314 CFS and lysates samples to have lactate concentration comparable to that of the lactic acid producing probiotics.

#### 3.2.11 *B. coagulans* CGI314 antagonistic activity against skin, UTI, oral and gastrointestinal pathogens

A total of 29 known bacterial pathogens were investigated in this study, out of which *B. coagulans* CGI314 exhibited inhibition zones against 14 pathogens, including those that are involved in gastrointestinal, urinary tract (UTI), oral and common skin infections such as *S. enteritidis*, *E. coli*, *S. flexneri*, *C. jejuni*, *S. aureus*, *S. saprophyticus*, *S. epidermidis, S. warneri*, *C. albicans*, *S. mutans*, and *S. sobrinus* (Table 2; Supplementary Figure 2). Results from this study demonstrated a broad antimicrobial profile of *B. coagulans* CGI314.

**TABLE 2:** *B. coagulans* CGI314 antimicrobial activity against urinary tract, intestinal, oral, and common skin pathogens.

Category	Bacterial species	Zone of inhibition
Gastrointestinal	*Salmonella enteritidis* ATCC13076	+
	*Yersinia enterocolitica* DSM4780	–
	*Escherichia coli* ATCC25922	+
	*Listeria monocytogenes* DSM20600	–
	*Shigella flexneri* DSM4782	+
	*Campylobacter jejuni* DSM4688	+
	*Campylobacter coli* DSM4689	–
UTI	*Pseudomonas aeruginosa* DSM3227	–
	*Gardnerella vaginalis* DSM4944	–
	*Mobiluncus curtisii* DSM23059	–
Skin	*Cutibacterium acnes* DSM1897	–
	*Staphylococcus pseudintermedius* DSM21284	+
	*Staphylococcus saprophyticus* DSM20229	+
	*Staphylococcus epidermidis* DSM20044	+
	*Staphylococcus aureus* DSM1104	–
	*Staphylococcus aureus* RF122	+
	*Streptococcus agalactiae* DSM2134	+
	*Corynebacterium flavescens* DSM20296	–
	*Erysipelothrix rhusiopathiae* DSM5055	–
UTI/Skin	*Staphylococcus warneri* DSM20316	+
Oral	*Streptococcus mutans* DSM20523	+
	*Streptococcus sobrinus* DSM20742	+
	*Arcanobacterium haemolyticum* DSM22856	–
UTI / Skin / Oral	*Candida albicans* DSM3454	+
UTI / Oral	*Acinetobacter baumannii* DSM30007	–
Gastrointestinal / UTI / Oral	*Enterococcus faecalis* DSM20478	+
Other	*Streptococcus pyogenes* DSM20565	–
	*Streptococcus pneumoniae* DSM24048	–

All data presented is generated from this study.

#### 3.2.12 Immunomodulatory effect of *B. coagulans* CGI314 in human cell lines

**FIGURE 11:**
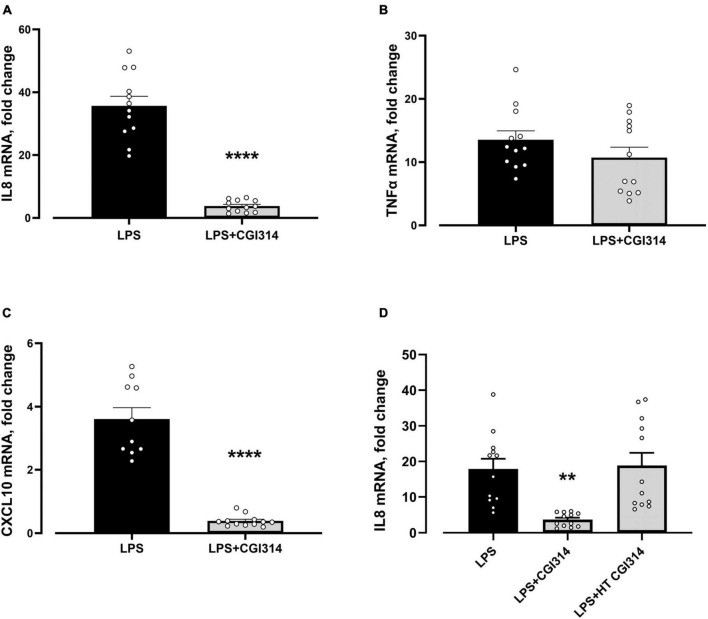
*Bacillus coagulans* CGI314 attenuated LPS-induced pro-inflammatory response in **(A)** IL-8 [*t*22 = 10.37, ****P < 0.0001] and **(C)** CXCL10 [*t*20 = 9.674, ****P < 0.0001], whereas no significant attentuation of LPS-induced pro-inflammatory response in **(B)** TNFα [*t*22 = 1.293, *P* = 0.2903] with CGI3141 in HT-29 cell line was seen. Heat treatment prevented the ability of *B. coagulans* CGI314 to attenuate LPS-induced increase in **(D)** IL-8 mRNA gene expression [*F*(2,33) = 10.01, *P* = 0.0004]. **** and ** represent a *post-hoc* significant difference with *p* < 0.0001 and *p* < 0.01, respectively.

The vegetative and heat killed form of *B. coagulans* CGI314 was assessed for its ability to modulate LPS-induced pro-inflammatory response in HT-29 cell line. The expression level of IL-8, TNF-α, and CXCL10 genes were evaluated at mRNA level using qRT-PCR. As shown in Figure 11 expression of all three genes were strongly induced 4 h after stimulation with LPS. Pretreatment with *B. coagulans* CGI314, but not with its heat -treated samples, greatly inhibited LPS-induced IL8 and CXCL10 gene expression in HT-29 cells.

#### 3.2.13 *B. coagulans* CGI314 increase the expression of tight junction proteins

**FIGURE 12:**
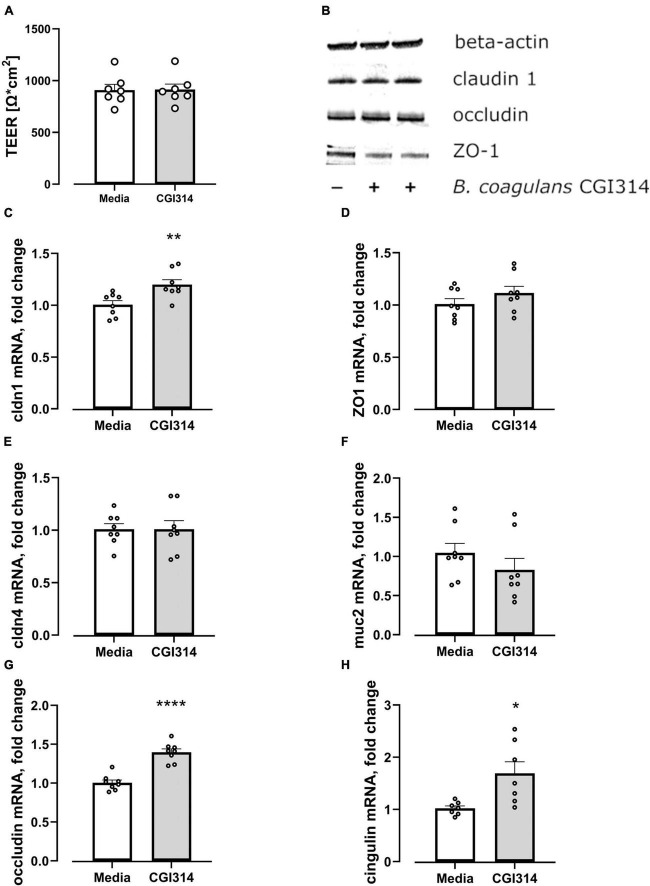
**(A)** *B. coagulans* CGI314 had no effect on the transepithelial electrical resistance (TEER) of HT-29 MTX epithelial cells *t*12 = 0.05863, *p* = 0.9542. **(B)** *B. coagulans* CGI314 had no significant effect on protein expression of tight junction proteins as determined by Western Blots. **(C–H)** *B. coagulans* CGI314 significantly increased gene expression of claudin 1 (*t*14 = 3.194, *p* = 0.0065), occludin (*t*14 = 6.920, *P* < 0.0001) and cingulin (*t*12 = 2.944, *P* = 0.0123) while having no effect on zonulin 1 (*t*14 = 1.284, *P* = 0.22), claudin 4 (t14 = 0.01135, *P* = 0.9911) and mucin 2 (t14 = 1.146, *P* = 0.2712). ****, ** and * represents a significant difference between *B. coagulans* CGI314 and media control with *p* < 0.0001, *p* < 0.01, and *p* < 0.05, respectively, as determined by *t*-test.

*Bacillus coagulans* CGI314 was assessed for the ability to impact intestinal-epithelial integrity by apically exposing HT-29-MTX cells to *B. coagulans* CGI314 for 20 h coupled with TEER measurements carried out before and after co-incubation with bacteria. There was no effect of *B. coagulans* CGI314 on barrier integrity alone suggesting it is not negatively impacting epithelial barrier (Figure 12A). Next, to assess whether *B. coagulans* CGI314 can influence the expression of genes coding for tight junctions (TJ) proteins, the expression of muc 2 gene coding for an intestinal mucin protein in addition to the tight junctions (TJ) proteins were evaluated. Co-incubation with *B. coagulans* CGI314 upregulated mRNA gene expression for claudin 1, occludin, and cingulin in HT-29-MTX. Expression levels of ZO-1, claudin 4, and muc2 genes remained unchanged (Figures 12C–H). Western blot did not report any significant change in protein expression of claudin 1, occluding or zonulin-1 (Figure 12B). Together, indicating no deleterious effect of CGI314 on barrier integrity.

#### 3.2.14 *B. coagulans* CGI314 attenuate H_2_O_2_-induced deficits in intestinal-epithelial barrier integrity

**FIGURE 13:**
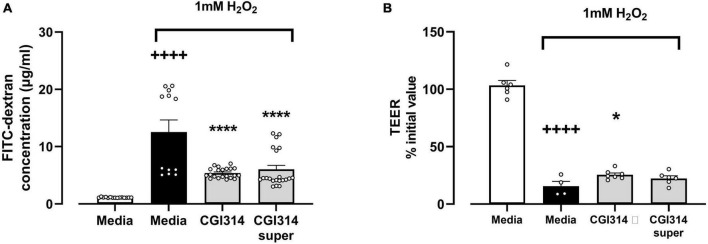
1mM H_2_O_2_ significantly increased **(A)** gut barrier permeability *t*25 = 6.096, ^++++^*P* < 0.0001 and **(B)** decreased trans-epithelial electrical resistance *t*8 = 14.19, ^++++^*P* < 0.0001 in HT-29 epithelial cell lines. *B. coagulans* CGI314 significantly attenuated the H_2_O_2_-induced increase in **(A)** gut barrier permeability [*F*(2,51) = 42.91, ****P < 0.0001] and **(B)** TEER [*F*(2,15) = 1.811, *P = 0.0459] as determined using one way ANOVA. ++++ represents a significant difference between control and H2O2-treated media groups. **** and * represents a significant difference with *p* < 0.0001 and *p* < 0.05, respectively, as determined by Dunnett’s *post-hoc* test as compared with H2O2-treated media control.

The effect of *B. coagulans* CGI314 and its cell free supernatants on the H_2_O_2_-induced disruption of intestinal epithelial barrier was further assessed. A significant reduction in TEER and increased dextran permeability was observed following treatment of differentiated HT-29-MTX cell monolayers with 1mM H_2_O_2_. Pre-incubation of epithelial cells with *B. coagulans* CGI314 partially attenuated the H_2_O_2_-induced decrease in TEER (Figure 13A) and increased flux of dextran (Figure 13B). Pre-treatment of cells with cell free supernatants was partially protective only against the effect of H_2_O_2_ on dextran permeability (Figure 13B). These findings suggest *B. coagulans* CGI314 protective effects against challenges against intestinal-epithelial barrier integrity.

## 4 Discussion

The key components of strains to qualify as probiotic candidates in foods and dietary supplements includes sufficient safety, identity, and functional characterization (Binda et al., 2020). Therefore, in this current study a combination of enzymatic, metabolomic and cell-based assays are employed to gain a better understanding of the functional and safety characteristics of the novel strain *B. coagulans* CGI314.

In respects to safety, the susceptibility and resistance of strains to commonly used antibiotics should be determined (EFSA, 2012; Ren et al., 2014). *B. coagulans* CGI314 was susceptible to eight antibiotics (Supplementary Table 1) recommended by European Food Safety Authority, using the MICs suggested for the *Bacillus* spp (Rychen et al., 2018). *B. coagulans* CGI314 was also negative for hemolytic activity on 5% (v/ v) sheep blood agar (Figure 1). A lack of hemolytic activity is regarded as an important criterion during the selection of probiotic strains, as hemolysin is an important virulence factor, and is more commonly associated with strains causing extraintestinal infections (Elliott et al., 1998). These findings align with previous reports demonstrating that 31 *B. coagulans* strains isolated from rice straw do not demonstrate hemolytic activity using 5% (v/ v) sheep blood agar (Kim et al., 2021).

We further assessed *B. coagulans* CGI314 cytotoxic potential on human colorectal adenocarcinoma cell line HT-29 and hepatocellular carcinoma cells line HepG2. No significant cytotoxic effects were observed with CGI314 on HT-29 and HepG2 cell lines using the XTT assay (Figure 2). Taken together these data suggest that *B. coagulans* CGI314 is safe and can be considered as a potential candidate for probiotic assessment.

A fundamental characteristic of probiotics is the ability to survive GI transit and reach the small intestine, where they colonize and impart positive health benefits to the host (Yasmin et al., 2020; Mazhar et al., 2023). The decrease in the viability and survival rates of several probiotic candidate strains can occur at this stage, due to the low pH (<3) and high concentrations of pepsin enzymes in gastric juice (Casarotti and Penna, 2015; Wendel, 2022). *B. coagulans* CGI314 spores demonstrate excellent resistance to the simulated gastric and small intestinal environment, indicating their potential ability to survive passage through upper gastrointestinal tract (Figure 4). Furthermore, *in vivo* models have shown that *Bacillus* spores including *B. coagulans* can germinate and proliferate in the GIT (Cao et al., 2020; Colom et al., 2021). Additionally, the ability of *B. coagulans* CGI314 to withstand different pasteurization temperatures *in vitro* at 45°C, 75°C and 90°C was investigated to assess stability during the manufacturing processes. *B. coagulans* CGI314 demonstrated superior stability over *L. rhamnosus* GG and *L. fermentum* ME-3 (Figure 3). These findings are in line with previous data investigating the stability of other members of *Bacillus* species in different food and beverage with relevant temperature stability testing (Khokhlova et al., 2023; Mazhar et al., 2023).

Adhesion and potential penetration of the mucus layer is another important probiotic attribute (Gorreja and Walker, 2022), as stable binding to the intestinal epithelium may be important for probiotic replication, colonization, immunomodulation, and inhibition of pathogen colonization. CGI314 spores showed higher adhesive capacity in the mucous-secreting HT-29-MTX cell line (19.6%) than HT-29 cells (0.9%) (Figure 5). However, in both, cell lines the adherence of CGI314 spores was lower than that of the reference probiotic strain *L. fermentum* GG. Nonetheless, CGI314 spores demonstrated moderate activity in adhering to the gastrointestinal epithelial cell lining. In addition, to adhering to host cells, the surface of the bacterial cells (fimbriae, adhesins) and secreted macromolecules such as exopolysaccharides, proteins, extracellular DNA and lipids allow bacteria to bind to themselves (Walter et al., 2008; Zawistowska-Rojek et al., 2022). This self-binding termed auto-aggregation is important for colonization of different surfaces, playing an important role in bacterial survival and may affect the longer duration of microorganisms in the digestive tract (Rajab et al., 2020). According to previous reports, a good auto-aggregation ability is reported to be greater than 40%, and any strain with less than 10% is considered to have weak auto-aggregation (Wang et al., 2010; Khalil et al., 2018). *B. coagulans* CGI314 was regarded to have moderate auto-aggregation ability with a value of 25.8% (Supplementary Figure 1; Supplementary Table 2). As co-aggregation traits are important criteria for food preservation and have an impact on eliminating pathogens (Xu et al., 2009), the ability of CGI314 to co-aggregate with pathogens such as *E. coli*, *E. faecalis*, and *S. aureus* strains was investigated. CGI314 displayed the ability to co-aggregate all the three test strains, with highest co-aggregation of 21.85% with *E. faecalis* DSM20478. Together, adhesion, auto-aggregation, and co-aggregation are properties that may provide a great advantage for microbial colonization in the intestinal tract (Pan et al., 2017).

In addition to aforementioned key probiotic attributes, further desirable probiotic functional properties include antioxidant, beneficial metabolite production (amino acids, vitamins, organic acids, short chain fatty acids, etc.) cholesterol assimilation, anti-inflammatory and antagonistic activities to confer positive health benefits to the host.

Oxidative stress is defined as a condition in which the prooxidant–antioxidant balance in the cell is disturbed, resulting in oxidative damage to lipids, proteins, DNA, ultimately compromising cell viability (Wang et al., 2017). Our bodies normally regulate oxidative stress through natural endogenous and dietary antioxidants. However, the efficiency of this protective physiological process can decrease as we age, or in times of stress or illness (Cui et al., 2012; Pizzino et al., 2017). Herein, we determined that *B. coagulans* CGI314 demonstrated superior levels of antioxidant enzyme and total antioxidant capacity over *L. rhamnosus* GG (Figure 6). Furthermore, the antioxidant activity of CGI314 and its cell free supernatant (CFS) was demonstrated in an assay of H_2_O_2_-induced ROS production and cell death (Figure 7). Together, these results suggest strong antioxidant capacity of *B. coagulans* CGI314.

The metabolic activity of *B. coagulans* CGI314 was assessed using commercial biochemical kits (proteases, peptidases, esterase’s, lactate, and carbohydrate fermentation), coupled with metabolomic analysis of fermented TSB broth and UHT milk. *B. coagulans* CGI314 was able to ferment 22 out of the 49 carbohydrates tested and was positive for esterase-lipase C8 and C4, leucine arylamidase, valine arylamidase, and cystine arylamidase, acid phosphatase, alkaline phosphatase and phosphohydrolase, α -galactosidase, α -glucosidase, β-galactosidase, and β-glucosidase activity. Bioactive capacities of lactate have been reported previously (Garrote et al., 2015), and CGI314 in its vegetative form has demonstrated to produce significant amounts of lactate from the fermentation of MRS (9.6 umol/ ml) broth in comparison to the TSB (2.09 umol / ml). In general, the amount of lactate produced in MRS broth was comparable to that of the concentration produced by *L. rhamnosus* GG, a positive control strain (Figure 10). Furthermore, CGI314 demonstrated the potential to synthesize riboflavin (vitamin B2) and folic acid (vitamin B9) in TSB broth (Figure 8). CGI314 also produced four essential amino acids in both, TSB and UHT milk (lysine, leucine, methionine, and tryptophan) and organic acids such as lactic acid, succinic acid and benzoic acid in UHT milk (Figure 8). Organic acids have been reported to be useful starting materials in multiple applications which includes food, cosmetic, chemical, pharmaceutical, and beverage industries owing to their various functional properties. Lactic acid, succinic acid and benzoic acid in food have been linked to flavor formation, antimicrobial and antioxidant effects (Florou-Paneri et al., 2013; Majchrzak et al., 2022; Punia Bangar et al., 2022; Nazareth et al., 2023). In UHT milk, *B. coagulans* CGI314 demonstrated the ability to synthesize the SCFAs; formic acid and acetic acid (Supplementary Table 3). SCFAs have been linked with beneficial effects in the host including anti-inflammatory, immunoregulatory, anti-obesity, anti-diabetes, anticancer, cardiovascular protective, hepatoprotective, and neuroprotective activities (Mirzaei et al., 2021; Nogal et al., 2021).

Excess cholesterol has been associated with several health implications, one of which is high risk of cardiovascular ailments (Ooi and Liong, 2010). Drugs used for the treatment of hypercholesterolemia have numerous side effects. Therefore, to avoid such effects, practicing dietary control or supplementation, especially with probiotics and/or prebiotics, has been investigated (Ooi and Liong, 2010; Tomaro-Duchesneau et al., 2014). *B. coagulans* CGI314 effectively reduced 6 mM cholesterol by 46.35% over 72 h, comparable with the positive control *L. rhamnosus* GG (Figure 9). Moreover, a time-dependent cholesterol reduction was observed, suggesting that the removal of cholesterol is correlated with the growth of CGI314. The results were in agreement with previous studies reporting the cholesterol removal properties of *Lactobacillus*, Bifidobacteria and other members of the *Bacillus* species (Pereira and Gibson, 2002; Wang et al., 2012; He et al., 2021).

It is important for probiotics to have antagonistic activities, which can include the production of antimicrobial compounds, competitive exclusion of pathogens, enhancement of the intestinal barrier function and others (Fijan, 2016). In literature, members of *B. coagulans* have demonstrated moderate antibacterial property due to the production of bacteriocins (Zhang et al., 2022). In the current study, a conventional phenotypic method (i.e., the double-layer agar method) was used to assess the antagonistic activity of CGI314. *B. coagulans* CGI314 exhibited antimicrobial activity against those pathogens involved in gastrointestinal, urinary tract, oral and common skin infections such as *S. enteritidis*, *E. coli*, *S. flexneri*, *C. jejuni*, *S. aureus*, *S. saprophyticus*, *S. epidermidis, S. warneri*, *S. mutans*, and *S. sobrinus* (Table 2; Supplementary Figure 1). Additionally, CGI314 displayed antagonistic activity toward *Candida albicans*, an opportunistic pathogenic yeast that is a common member of the human gut microbiota (Table 2). Considering all these findings, CGI314 has potential to control the presence of opportunistic pathogens in the gut, urinary tract, and mouth where semi-liquid to liquid conditions will be common. Furthermore, CGI314 also has the potentially to prevent the spread of opportunistic pathogens on dryer environments like the human skin. Altogether, the results from this study demonstrated a broad antimicrobial profile of CGI314, corroborating the antagonistic nature of the members of the *B. coagulans* as described in previous studies using other methods (Drago and De Vecchi, 2009; Cao et al., 2020; Sui et al., 2020).

Disruption to gut microflora, environmental factors such as stress, and diet, as well as genetic factors can contribute to inflammatory processes in the host (Liu et al., 2017; Al Bander et al., 2020; Khokhlova et al., 2023). The efficacy of *B. coagulans* CGI314 in ameliorating LPS-induced increase in inflammatory tone was assessed *in vitro*. Pretreatment of HT-29 cells with vegetative *B. coagulans* CGI314 attenuated the LPS-induced expression of pro-inflammatory (IL-8 and CXCL10) genes (Figure 11) suggesting a potential immunomodulatory role. These findings support other studies with *B. coagulans* strains demonstrating antioxidant and immunomodulatory potential in an LPS-induced caecal injury model in rats (Wang et al., 2022).

The protective effect of *B. coagulans* CGI314 on barrier integrity was investigated using Real-Time Quantitative Reverse Transcription PCR (RT- qPCR) and western blotting techniques for assessing tight junction protein expression including occludin, claudins, and zonula occludens-1 (ZO-1) associated with intestinal epithelial barrier integrity. *B. coagulans* CGI314 caused an upregulation of claudin 1, occludin, and cingulin mRNA alone with no changes in mRNA gene expression for ZO-1, claudin 4 and muc2 genes (Figure 12). However, *B. coagulans* CGI314 did not significantly increase the protein expression of these tight junction proteins as determined by Western blot (Figure 12B). We further demonstrated that *B. coagulans* CGI314 partially reduced the 1mM H_2_O_2–_induced decrease in TEER (Figure 13A) in addition to increased flux of dextran (Figure 13B). While cell free supernatants of CGI314 were partially protective against the effect of 1mM H_2_O_2_ on dextran permeability (Figure 13B). Overall, the data from these assays indicate the potential immunomodulatory and intestinal barrier protective properties of *B. coagulans* CGI314.

Altogether, these findings suggest that CGI314 has numerous desirable traits of a potential probiotic in functional food including thermostability, high antioxidant, antagonistic and immunomodulatory capacity, diverse carbohydrate fermentation profile with the ability to ferment rich medias and produce bioactive compounds (vitamins, amino acid and organics acids), cholesterol lowering ability and potential to modulate and improve intestinal epithelium permeability.

## 5 Conclusion

The study presented an extensive overview of the potential probiotic properties of the novel strain of *B. coagulans* CGI314, starting with the safety of this strain using conventional safety evaluation approaches, including hemolysis, antimicrobial resistance and *in vitro* cell based models to assess cytotoxicity. *B. coagulans* CGI314 was non-hemolytic, non-cytotoxic, and was observed to be sensitive to all the antibiotics recommended by EFSA, which are desirable traits as most of the antibiotics tested belongs to the major classes of antibiotics used for human clinical treatments. Regarding probiotics attributes, *B. coagulans* CGI314 displayed strong thermostability and high level of resistance to gastric and intestinal conditions when compared with member of the lactic acid bacteria. Additionally*, B. coagulans* CGI314 displayed moderate adhesion capacity in mucus-secreting HT29-MTX intestinal cell models, and displayed a broad antimicrobial activity against those that are involved in urinary tract, intestinal, oral, and common skin infections suggesting, *B. coagulans* CGI314 has the potential in controlling or preventing the presence of such opportunistic pathogens. Quantitative analysis of *B. coagulans* CGI314 total antioxidant, catalase, and reduced glutathione (GSH) activities were superior to the well-researched control strain *L. rhamnosus* GG. Antioxidant activities of *B. coagulans* CGI314 were further characterized using human *in vitro* cell models (HepG2 and HT29), where *B. coagulans* CGI314 acted on ROS by lowering the oxidative damage induced by H_2_O_2_. Cholesterol assimilation and lactate production in media by *B. coagulans* CGI314 was comparable to that of the control strain *L. rhamnosus* GG. Additionally, *B. coagulans* CGI314 exhibited strong ability to attenuate LPS triggered inflammatory response in the intestinal epithelial cell line and partially ameliorated the detrimental effects of H_2_O_2_-induced intestinal epithelial barrier integrity. Fermentation of rich medias (TSB and UHT milk) with this novel strain displayed the production of a several amino acids, some water soluble vitamins (B2 and B9) and SCFAs (formic acid and acetate). Combined with its carbohydrate assimilation profile, the potential ability of this strain to promote the absorption and utilization of dietary proteins and carbohydrates as well as possibly synthesizing bioactive compound including essential amino acid, vitamins, SCFAs and organic acids represent some intriguing properties. Together, findings from this study provide strong evidence of the probiotic potential of *B. coagulans* CGI314 which merits further investigation of this strain as a candidate probiotic in food products.

## Data availability statement

The original contributions presented in this study are included in this article/Supplementary material, further inquiries can be directed to the corresponding author.

## Ethics statement

Ethical approval was not required for the studies on animals in accordance with the local legislation and institutional requirements because only commercially available established cell lines were used.

## Author contributions

SM: Data curation, Formal analysis, Investigation, Methodology, Validation, Writing—original draft. AS: Data curation, Formal analysis, Investigation, Methodology, Validation, Writing—review and editing. EK: Data curation, Formal analysis, Investigation, Methodology, Validation, Writing—review and editing. JC: Conceptualization, Investigation, Project administration, Resources, Validation, Writing—review and editing. NL: Data curation, Formal analysis, Investigation, Methodology, Validation, Writing—review and editing. JD: Funding acquisition, Project administration, Supervision, Writing—review and editing. KR: Conceptualization, Funding acquisition, Project administration, Resources, Supervision, Writing—review and editing.
