# Supplementary material for: In vitro safety and functional characterization of the novel Bacillus coagulans strain CGI314
Source: Front Microbiol. 2024 Jan 10;14:1302480. doi: 10.3389/fmicb.2023.1302480 (PMC10809412; doi:10.3389/fmicb.2023.1302480)
Supplement: Supplementary file 1 [file Data_Sheet_1.docx]

Supplementary Material

**Table S1.** Minimum Inhibitory Concentration (MIC) of antibiotics against *B. coagulans* CGI314. All data presented is generated from this study.

| **Antibiotic** | **MIC (mg/ L)** |
| --- | --- |
|  | ***B. coagulans* CGI314** |
| Vancomycin | < 0.125 |
| Gentamicin | < 0.125 |
| Kanamycin | < 0.125 |
| Streptomycin | < 0.25 |
| Erythromycin | < 4.0 |
| Clindamycin | < 0.125 |
| Tetracycline | < 0.25 |
| Chloramphenicol | < 4.0 |

MICs of antibiotics were determined as per CLSI guidelines against *B. coagulans* CGI314

**Table S2**. Auto-aggregation and Co- aggregation of CGI314 and pathogenic strains after 24h incubation. Average value and ± SD from 3 technical replicates.

| **Strain** | **% Auto-aggregation** | **SD** |
| --- | --- | --- |
| **Fortispore CGI314** | 25.85 | 0.01 |
| ***E. coli* ATCC25922** | 0.11 | 0.01 |
| ***E. faecalis* DSM20478** | 1.26 | 0.05 |
| ***S. aureus* DSM17091** | 21.42 | 0.02 |
|  | **% Co-aggregation** | **SD** |
| **Fortispore (CGI314) + *E. coli* ATCC25922** | 10.52 | 0.01 |
| **Fortispore (CGI314) + *E. faecalis* DSM20478** | 21.85 | 0.01 |
| **Fortispore (CGI314) + *S. aureus* DSM17091** | 20.40 | 0.01 |

**Table S3**. List of compounds significantly associated with fermentation of UHT milk and TSB media by CGI314 analyzed with GC-MS, HPLC and UHPLC based methods. Mean values of compounds identified in CGI314 and Control (uninoculated) UHT milk or TSB.

|  |  |  | **Relative Concentration** |  |
| --- | --- | --- | --- | --- |
| **Vitamins** | **Compound** | **pValue** | **Mean of control** | **Mean of CGI314** |
| TSB | Thiamine (B1) | 0.000004 | 0.4333 | 0 |
|  | Riboflavin (B2) | 0.007763 | 0.2333 | 0.2567 |
|  | Nicotinic acid (B3) | 0.00001 | 0.3533 | 0.02 |
|  | Pantothenic acid (B5) | 0.260575 | 0.01 | 0.03 |
|  | Pyridoxine (B6) | 0.000017 | 3.297 | 0.03333 |
|  | Biotin (B7) | 0.373901 | 0 | 0.03667 |
|  | Folic acid (B9) | 0.000065 | 0 | 0.9067 |
|  | Cyanocobalamin (B12) | 0.507158 | 0.03667 | 0.02667 |
| **Amino acids** | Aspartate | 0.480011 | 31.29 | 24.7 |
| TSB | Serine + Asparagine | 0.22494 | 97.17 | 82.4 |
|  | Glutamic acid | 0.624043 | 109.6 | 88.2 |
|  | Glycine | 0.00032 | 46.38 | 31.61 |
|  | Glutaminylhistidine | 0.00085 | 37.26 | 129.6 |
|  | Arginine | 0.000316 | 389 | 134 |
|  | Threonine | 0.038731 | 59.66 | 44.92 |
|  | Alanine | 0.000109 | 65.78 | 6.037 |
|  | Proline | 0.243816 | 13.26 | 18.19 |
|  | Cysteine | 0.540681 | 56.53 | 54.59 |
|  | Tyrosine | 0.000198 | 121.1 | 213.8 |
|  | Valine | 0.007132 | 107 | 155.5 |
|  | Methionine | 0.026505 | 85.67 | 111.1 |
|  | Lysine | 0.000828 | 596.1 | 356.1 |
|  | Isoleucine | 0.023192 | 101.1 | 71.89 |
|  | Leucine | 0.001721 | 428.1 | 251.4 |
|  | Phenylalanine | 0.004261 | 250.1 | 174.2 |
|  | Lysine | 0.000045 | 0.01 | 0.12 |
| UHT Milk | Histidine | 0.00415 | -0.01 | 0.08 |
|  | Tryptophan | 0.001324 | 0 | 0.02667 |
|  | Cystine | 0.2302 | -0.01333 | -0.006667 |
|  | Ornithine | >0.999999 | 0.03667 | 0.03667 |
|  | Serine | 0.084204 | 0.27 | 0.9 |
|  | Pyruvic acid | 0.007759 | -0.05667 | 0.5867 |
|  | Fumaric acid | 0.053699 | -0.4967 | -0.1933 |
|  | Succinic acid | 0.000068 | 0.11 | 2.143 |
|  | Lactic acid | 0.000357 | 0.01 | 1.73 |
|  | 2-Oxoglutaric acid | 0.001548 | 0.7567 | -0.02333 |
|  | 2-Phosphoenolpyruvic acid | >0.999999 | -0.03667 | -0.03667 |
|  | Malic acid | 0.062296 | 1.577 | 1.14 |
|  | cis-Aconitic acid | 0.004148 | 1.133 | 1.647 |
|  | Citric acid | 0.01261 | 1.557 | 1.383 |
|  | Isocitric acid | 0.000349 | 0.8733 | 1.61 |
|  | 4-methyl-2-oxopentanoic acid | 0.108262 | -0.03333 | 0.02333 |
|  | 3-(methylthio)propionic acid | >0.999999 | 0.04 | 0.04 |
|  | Octanoic acid | 0.260778 | 1.453 | 1.29 |
|  | 3-Methyl-oxirane-2-carboxylic acid | 0.000306 | -0.09 | 0.5333 |
|  | Benzoic acid | 0.000125 | 0.03333 | 1.603 |
|  | Phenylacetic acid | 0.01613 | -0.003333 | 0.01 |
|  | Decanoic acid | 0.100388 | 1.623 | 1.3 |
|  | Dodecanoic acid | 0.215656 | 1.733 | 1.427 |
|  | Tetradecanoic acid | 0.229633 | 2.637 | 2.177 |
|  | Alanyl-proline | 0.000197 | -0.05667 | 0.06667 |
|  | Glycyl-proline | 0.013236 | -0.01333 | 0.02667 |
|  | 5-Aminovaleric acid | 0.017773 | 1.377 | 1.16 |
| **SCFA** | Acetic acid | 0.001296 | 0.1333 | 0.61 |
| UHT Milk | Formic acid | 0.001594 | 0.06333 | 1.107 |
|  | Propanoic acid | 0.773473 | 0.2567 | 0.2867 |
|  | 2-methyl-propanoic acid | 0.648221 | 0.1667 | 0.13 |
|  | Butanoic acid | 0.529703 | 0.1767 | 0.1167 |
|  | 3-methylbutanoic acid | 0.87433 | 0.1267 | 0.11 |
|  | Pentanoic acid | 0.369038 | 18813993 | 20911978 |
|  | 4-methyl-pentanoic acid | 0.2302 | 0.01333 | 0.006667 |
|  | Hexanoic acid | 0.188597 | 0.37 | 0.2 |
|  | Heptanoic acid | >0.999999 | 0.006667 | 0.006667 |

**Table S4.** Carbohydrate fermentation profile of CGI314 using API 50 CH kit system. All tests were performed in triplicate. All results presented are generated from this study. +, Positive; -, Negative, and (+) Variable.

|  |  |  |  |
| --- | --- | --- | --- |
| **Carbohydrate** | ***-/+*** | **Carbohydrate** | ***-/+*** |
| Glycerol | - | Salicin | + |
| Erythritol | - | D-cellobiose | + |
| D-arabinose | - | D-maltose | + |
| L-arabinose | + | D-lactose | - |
| D-ribose | + | D-melibiose | - |
| D-xylose | + | D-saccharose | - |
| L-xylose | - | D-trehalose | + |
| D-adonitol | - | Inulin | - |
| Methyl-bd-xylopyranoside | - | D-melezitose | - |
| D-galactose | + | D-rafinose | - |
| D-glucose | + | Amidon | + |
| D-fructose | + | Glycogen | - |
| D-mannose | + | Xylitol | - |
| L-sorbose | - | Gentiobiose | + |
| L-rhamnose | - | D-turanose | - |
| Dulcitol | - | D-lyxose | - |
| Inositol | - | D-tagatose | - |
| D-mannitol | + | D-fucose | - |
| D-sorbitol | + | L-fucose | - |
| Methyl-ad-mannopyranoside | - | D-arabitol | (+) |
| Methyl-ad-glucopyranoside | + | L-arabitol | - |
| N-acetylglucosamine | + | Pottasium gluconate | + |
| Amygdalin | + | Pottasium 2-ketogluconate | - |
| Arbutin | + | Pottasium 5-ketogluconate | - |
| Esculin ferric citrate | + |  |  |

**Table S5.** Semi-Quantitative assay of enzyme activities of CGI314. All tests were performed in triplicates. All results presented are generated from this study. +, Positive; -, Negative.

| **Enzyme** | | ***B. coagulans* CGI314** |
| --- | --- | --- |
| Esterase activity | Esterase (C4:0) | + |
|  | Esterase (C8:0) | + |
| Lipase activity | Lipase (C14:0) | - |
| Peptidase activity | Leucine arylamidase | + |
|  | Valine arylamidase | + |
|  | Cystine arylamidase | + |
| Proteinase activity | Trypsin | - |
|  | α-chymotrypsin | - |
| Phosphatase activity | Acid phosphatase | + |
|  | Alkaline phosphatase | + |
|  | Phosphohydrolyase | + |
| Glycosidase activity | α-Galactosidase | + |
|  | β-Galactosidase | + |
|  | β-Glucuronidase | - |
|  | α-Glucosidase | + |
|  | β-Glucosidase | + |
|  | β-Glucosaminidase | - |
|  | α-Mannosidase | - |
|  | α-Fucosidase | - |

**Table S6.** Primers used in this study

| **Primer** | **Sequence** | **Product Length** |
| --- | --- | --- |
| gapdh-F | CTTTGACGCTGGGGCTGGCATT | 161 |
| gapdh-R | TTGTGCTCTTGCTGGGGCTGGT |  |
| IL-8-F | CAGTTTTGCCAAGGAGTGCT | 198 |
| IL-8-R | CAACCCTCTGCACCCAGTTT |  |
| TNF-alpha-F | GCCAGAGGGCTGATTAGAGA | 82 |
| TNF-alpha-R | TCTTCTGCCTGCTGCACTT |  |
| cxcl10-F | TTCAAGGAGTACCTCTCTCTAG | 177 |
| cxcl10-R | CTGGATTCAGACATCTCTTCTC |  |
| homo-ZO-1-F | CGGGACTGTTGGTATTGGCTAGA | 184 |
| homo-ZO-1-R | GGCCAGGGCCATAGTAAAGTTTG |  |
| homo-Claudin-1-F | GCGCGATATTTCTTCTTGCAGG | 113 |
| homo-Claudin-1-R | TTCGTACCTGGCATTGACTGG |  |
| OCLN-F | CGTCAGGTGCGCCCGCCAG | 113 |
| OCLN-R | GAGCAATGCCCTTTAGCTTCC |  |
| CGN-F | CGAACGCAAGCCTGGGAG | 106 |
| CGN-R | CTGGACTCCATGGTCTACGG |  |
| homo-Muc-2-F | AACGGCCTGCAGAGCTATTC | 80 |
| homo-Muc-2-R | ATCTTCTGCATGTTCCCAAACTC |  |

**Table S7.** Antibodies used in this study

|  | Dilution of primary antibodies | Dilution of secondary antibodies (Cat#AS014) | AB clonal cat# (primary antibodies) |
| --- | --- | --- | --- |
| β-actin/polyclonal pre-diluted | 1:500 | 1:2,000 | AC026 |
| claudin 1/monoclonal | 1:500 | 1:2,000 | A11530 |
| occludin/polyclonal | 1:2000 | 1:5,000 | A12621 |
| ZO-1/polyclonal | 1:1000 | 1:5,000 | A0659 |

**Figure S1**. (A) Auto-aggregation of CGI314 and pathogenic bacteria over 24hr incubation. Average value and ± SD from 3 technical replicates. (B) Co-aggregation of CGI314 with pathogenic bacteria over 24hr incubation. Average value and ± SD from 3 technical replicates.


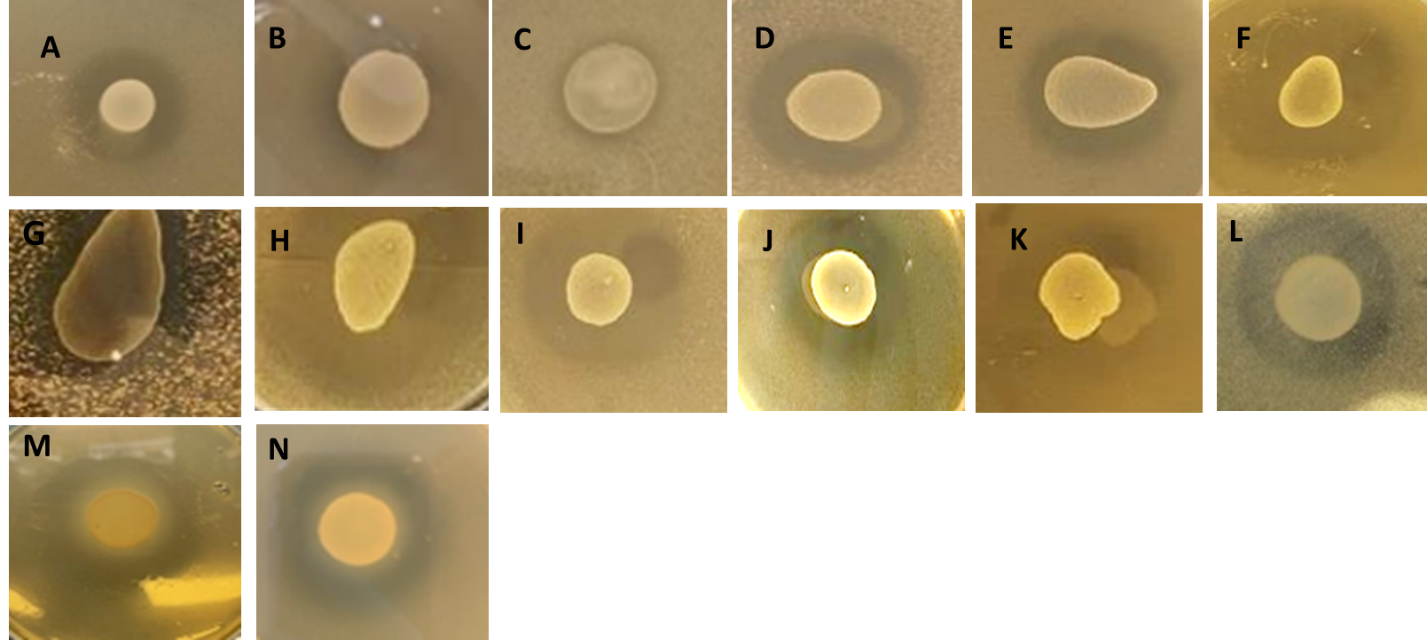


**Figure S2.** Antimicrobial activity of *B. coagulans* CGI314 against urinary tract, intestinal, oral and common skin pathogens. (A) *Escherichia coli* ATCC25922, (B) *Salmonella enteritidis* ATCC13076, (C) *Staphylococcus aureus* RF122, (d) *Staphylococcus warneri* DSM20316, (E) *Staphylococcus epidermidis* DSM20044, (F) *Shigella flexnerii* DSM4782, (G) *Candida albicans* DSM3454, (H) *Staphylococcus pseudintermedius* DSM21284, (I) *Staphylococcus saprophiticus* DSM20229, (J) *Enterococcus faecalis* DSM20478, (K) *Streptococcus agalactiae* DSM2134, (L) *Campilobacter jejuni* DSM4688, (M) *Streptococcus mutans* DSM 20523^T^ , and (N) *Streptococcus sobrinus* DSM 20742^T^.
